# Clinical, developmental and serotonemia phenotyping of a sample of 70 Italian patients with Phelan-McDermid Syndrome

**DOI:** 10.1186/s11689-024-09572-7

**Published:** 2024-10-03

**Authors:** Lisa Asta, Arianna Ricciardello, Francesca Cucinotta, Laura Turriziani, Maria Boncoddo, Fabiana Bellomo, Jessica Angelini, Martina Gnazzo, Giulia Scandolo, Giulia Pisanò, Francesco Pelagatti, Fethia Chehbani, Michela Camia, Antonio M. Persico

**Affiliations:** 1 Department of Biomedical, Metabolic and Neural Sciences, University of Modena and Reggio Emilia, Modena, Italy; 2 Cantonal Psychiatric Clinic, Cantonal Socio-Psychiatric Organization (O.S.C.), Repubblica e Cantone Ticino, Mendrisio, Switzerland; 3 IRCCS Centro Neurolesi “Bonino-Pulejo”, Messina, 98124 Italy; 4 Center for Autism “Dopo Di Noi”, Barcellona Pozzo Di Gotto (Messina), Italy; 5 Institute for Biomedical Research and Innovation (I.R.I.B.), National Research Council of Italy (C.N.R.), Messina, Italy; 6 Child Neuropsychiatry Unit, “G. Martino” University Hospital, Messina, Italy; 7 Residency Program in Child & Adolescent Neuropsychiatry, University of Modena and Reggio Emilia, Modena, Italy; 8 Child & Adolescent Neuropsychiatry Program, Modena University Hospital, Modena, Italy

**Keywords:** 22q13 deletion syndrome, Autism spectrum disorder, Hyperserotonemia, Intellectual disability, Macrocephaly, Neuroinflammation, Phenotype, Phelan-McDermid syndrome, Serotonin, *SHANK3*

## Abstract

**Background:**

Phelan-McDermid syndrome (PMS) is caused by monoallelic loss or inactivation at the *SHANK3* gene, located in human chr 22q13.33, and is often associated with Autism Spectrum Disorder (ASD).

**Objectives:**

To assess the clinical and developmental phenotype in a novel sample of PMS patients, including for the first time auxometric trajectories and serotonin blood levels.

**Methods:**

70 Italian PMS patients were clinically characterized by parental report, direct medical observation, and a thorough medical and psychodiagnostic protocol. Serotonin levels were measured in platelet-rich plasma by HPLC.

**Results:**

Our sample includes 59 (84.3%) cases with chr. 22q13 terminal deletion, 5 (7.1%) disruptive *SHANK3* mutations, and 6 (8.6%) ring chromosome 22. Intellectual disability was present in 69 (98.6%) cases, motor coordination disorder in 65 (92.9%), ASD in 20 (28.6%), and lifetime bipolar disorder in 12 (17.1%). Prenatal and postnatal complications were frequent (22.9%-48.6%). Expressive and receptive language were absent in 49 (70.0%) and 19 (27.1%) cases, respectively. Decreased pain sensitivity was reported in 56 (80.0%), hyperactivity in 49 (80.3%), abnormal sleep in 45 (64.3%), congenital dysmorphisms in 35 (58.3%), chronic stool abnormalities and especially constipation in 29 (41.4%). Parents reported noticing behavioral abnormalities during early childhood immediately after an infective episode in 34 (48.6%) patients. Brain MRI anomalies were observed in 53 (79.1%), EEG abnormalities in 16 (23.5%), kidney and upper urinary tract malformations in 18 (28.1%). Two novel phenotypes emerged: (a) a subgroup of 12/44 (27.3%) PMS patients displays smaller head size at enrollment (mean age 11.8 yrs) compared to their first year of neonatal life, documenting a deceleration of head growth (*p* < 0.001); (b) serotonin blood levels are significantly lower in 21 PMS patients compared to their 21 unaffected siblings (*P* < 0.05), and to 432 idiopathic ASD cases (*p* < 0.001).

**Conclusions:**

We replicate and extend the description of many phenotypic characteristics present in PMS, and report two novel features: (1) growth trajectories are variable and head growth appears to slow down during childhood in some PMS patients; (2) serotonin blood levels are decreased in PMS, and not increased as frequently occurs in ASD. Further investigations of these novel features are under way.

**Supplementary Information:**

The online version contains supplementary material available at 10.1186/s11689-024-09572-7.

## Introduction

Phelan McDermid syndrome (PMS), also known as 22q13 deletion syndrome (OMIM#606,232), is a rare genetic disorder with prominent neurodevelopmental components, mainly characterized by global developmental delay, intellectual disability, muscle hypotonia, severely impaired or absent speech, and minor congenital dysmorphisms [1, 2]. Early signs of PMS frequently include feeding difficulties, possibly due to muscle hypotonia [1–4]. An abnormal EEG with or without seizures [3–11], and brain MRI positive for thinning or hypoplasia of the corpus callosum, delayed myelination or other white matter anomalies, cerebellar hypoplasia, arachnoid cysts [3, 4, 8, 11–15] are also frequently observed. Microcephaly or, more often, macrocephaly have been occasionally reported [4, 6, 7, 12], although in most patients occipitofrontal head circumference seems to fall within the norm for age [16, 17]. Normal to accelerated growth has been reported in some [18, 19], but not all studies [16], although also the latter study found that the proportion of cases falling > 95th and < 3rd percentile was significantly different than expected by chance [16]. Other medical conditions frequently associated with PMS include sleep disturbances, lymphedema, gastroesophageal reflux, renal malformations, and immune deficits [4, 20–22].

The prevalence of PMS is estimated to be about 2.5–10 per million births [23], and currently more than 3000 patients worldwide are registered in the “Phelan-McDermid Syndrome International Registry” of the PMS Foundation [24]. However, this condition remains underdiagnosed, because of its nonspecific clinical manifestations and the need for genetic testing [2]. The neurobehavioral phenotype of PMS is typically caused by haploinsufficiency of the *SHANK3* gene [1, 21, 25], encoding a scaffolding protein located in the postsynaptic density of glutamatergic synapses, which plays a crucial role in the formation and management of dendritic spines and excitatory synapses [26]. *SHANK3* haploinsufficiency is usually due to a terminal or intragenic deletion of chr. 22q13, although *SHANK3* disruptive mutations are increasingly being discovered as next-generation sequencing (NGS) becomes more widespread in clinical settings [1, 3–5, 9, 12, 27]. Genotype–phenotype correlation studies support the association between deletion size and some clinical manifestations, such as developmental delay, dysmorphisms, medical comorbidities, hypotonia and communication abilities, with larger deletions generally yielding more severe phenotypes [4, 7, 9, 15, 22, 28–30], although exceptions have also been reported [15, 20]. Indeed, some manifestation frequently observed in PMS, such as renal abnormalities and lymphedema, are not linked to *SHANK3* haploinsufficiency, but depend on other 22q13 deleted genes, which play a role in shaping the PMS phenotype in each patient [15, 31, 32], The relevance of other genes in addition to *SHANK3* is further underscored by the existence of a phenotype consistent with PMS in individuals carrying interstitial 22q13 deletions that preserve *SHANK3*, leading to distinguish between PMS-SHANK3 related and PMS-SHANK3 unrelated forms [33].

*SHANK3* deficiency has been linked to several psychiatric conditions [34, 35], including autism spectrum disorder (ASD) [36–41], bipolar disorder [42–44], and schizophrenia [45, 46]. *SHANK3* deletions or disruptive mutations have been found in approximately 2% of cases diagnosed with ASD and intellectual disability [39], making it one of the common monogenic causes of ASD [4, 21]. Autistic symptoms are frequently observed in PMS patients [1, 3–5, 9, 20, 27], with studies reporting prevalence rates ranging from less than 30% [28] to more than 80% [4, 27], probably depending on the different diagnostic methodology employed across studies [4]. Some authors have suggested that the autistic phenotype observed in PMS may be different from idiopathic Autism Spectrum Disorder (iASD), as defined by DSM-5, at least concerning the repetitive and stereotyped behaviors domain [14, 27]. Philippe and Colleagues [14] described 8 children with 22q13.3 deletion syndrome with high ADI-R scores in the domains of social interactions, social communication and play, but not in repetitive and stereotyped pattern of behaviors, thus lacking one fundamental DSM-5 criterion for an ASD diagnosis. Similar results were reported by Oberman and Colleagues [27], who found anomalies in social communication almost in their entire sample (90%), but repetitive behaviors in approximately half (55%). Interestingly, in the latter study more participants showed sensory seeking activities (68%) [27]. More recently, one study [47] comparing repetitive behaviors in PMS and iASD found Repetitive Behavior Scales‑Revised (RBS‑R) total scores nearly half as high in the former sample, compared to the latter, suggesting that repetitive behaviors may be less intense and less widespread in PMS compared to iASD. Taken together, these findings suggest that autism profiles in PMS may be somewhat atypical and usually linked to developmental delay.

Anomalies in sensory processing have been recognized as a frequent characteristic of autistic individuals [48, 49], such that this feature has been included in DSM-5 criterion B for an ASD diagnosis [50]. Atypical sensory reactivity is frequently found also in PMS, especially decreased pain sensitivity, increased reactivity to tactile stimuli, and self-stimulatory behaviors [4, 7, 9, 19, 20, 22]. Nonetheless, also sensory profiles may differ between the two conditions, with PMS patients displaying more “low energy” symptoms and less hypersensitivity compared to autistic individuals [51]. Problem behaviors, including hyperactivity, attention deficits, aggressiveness toward self or others, and irritability, have been frequently observed in PMS [3, 4, 9, 14, 29], as well as blunted adaptive behaviors [1, 3, 9, 52, 53]. Several studies also report a regression of previously acquired cognitive and/or behavioral skills that may be preceded by triggering events, such as infections or seizures, and sometimes coincide with the onset of a psychiatric disorder [4, 5, 9, 54–56]. Usually this phenomenon affects language abilities, self-help skills, and motor abilities, with onset around mid-childhood [54, 56, 57], thus differentiating PMS from iASD, whereby regression, if present, occurs much earlier [57].

One of the best-established biomarkers of iASD is blood serotonin (5-hydroxytryptamine; 5-HT), whose levels are higher compared to controls in 22%-28% of autistic individuals [58]. Hyperserotonemia has been variably linked to social difficulties, language impairment, stereotypic and repetitive behaviors, absent or delayed social smile [59–62], among others. On the other hand, low serotonin blood levels have been associated with self-injurious behaviors, suicide attempts and depression [63, 64]. Since patients with PMS frequently display autistic features and PMS has been viewed as a monogenic form of ASD, we may expect to find elevated 5-HT blood levels in this population, but this hypothesis has not yet been investigated.

The aim of the present study is to provide a detailed description of the clinical, developmental, and auxometric phenotype of a new sample of 70 Italian patients genetically diagnosed with PMS. Blood samples were also collected to measure serotonin blood levels and compare PMS patients with their unaffected siblings.

## Methods

This study was approved by the Institutional Review Board of University ‘‘Campus Bio-Medico’’ of Rome, Italy (prot. n. 14/98, first approval on April 28, 1998 and subsequent amendments) and the Ethics Committee of Messina, Italy (prot. n. 22/17, approved on June 19, 2017). All parents gave written informed consent for themselves and for their affected offspring. All procedures performed in studies involving human participants are in accordance with the ethical standards of the institutional and/or national research committee and with the Helsinki declaration (2000).

### Participants

All patients with a genetically documented diagnosis of Phelan-McDermid Syndrome were consecutively enrolled in this study between 2013 and 2021, after reporting at the Campus Bio-Medico University Hospital (Rome, Italy), and at the Interdepartmental Program “Autism 0–90” of the “G. Martino” University Hospital of Messina (Italy) for medical follow-up and treatment. No exclusion criterion was applied. In particular, the presence of acute neuropsychiatric disorders or medical conditions was not a cause for exclusion, because their exact lifetime prevalence in PMS has not yet been conclusively determined.

### Assessment

Patients underwent a comprehensive medical evaluation, started as outpatients and then completed as inpatients, except for nine cases which were enrolled during the COVID pandemia and whose information was collected only via web. The assessment protocol included a thorough physical and neurological examination, routine hematology and blood chemistry including thyroid and liver function, EKG, cardiac ultrasound, wake/sleep EEG, abdominal ultrasound, ophthalmological visit, brain MRI (3 T), and auditory evoked potentials (Suppl. Figure 1A). In addition, optional consultations and exams were performed, as listed in Suppl. Figure 1A, depending on specific needs and clinical presentations. A detailed, family, developmental, medical and psychiatric history was collected from parents. A detailed mental status examination was performed, applying DSM-5 diagnostic criteria [50]. Chr. 22q13.3 deletion size was assumed valid if determined by CGH-array (at least Agilent 180 K or equivalent).

A complete evaluation of symptomatology, neuropsychological and behavioral skills was also carried out (Suppl. Figure 1B). Given the extended duration of recruitment for this study, only the most recent edition of each test and questionnaire is cited here. Autism Diagnostic Observation Schedule – 2 (ADOS-2) [65] and Autism Diagnostic Interview – Revised (ADI-R) [66] were administered for the evaluation of autistic symptomatology. ADOS-2 is a semi-structured standardized tool administered by a trained clinician to evaluate social affect and communication skills, and it also provides a total score. ADI-R is a semi-structured interview administered to caregivers and usually used together with ADOS-2. ADI-R provides information on (A) social interaction, (B) communication, and (C) restricted, repetitive, and stereotyped patterns of behavior, as well as on (D) anomalies in development before 36 months. Repetitive behaviors were also assessed with the Repetitive Behavior Scale – Revised (RBS-R) [67], while the Short Sensory Profile (SSP) [68] was used for the evaluation of sensory processing patterns in everyday situations. The Aberrant Behavior Checklist (ABC) [69] was administered to assess problematic behaviors, as well as the Child Behavior Checklist (CBCL) [70] and the parallel Teacher Report Form (TRF) [71]. Adaptive functioning was evaluated using the Vineland Adaptive Behavior Scale-II (VABS-II) [72], a standardized semi-structured parental interview to measure adaptive functioning in real life. Intellectual/developmental quotient were assessed using the Griffiths Scales of Child Development- III [73], Wechsler Intelligence Scale for Children—Fourth Edition (WISC-IV) [74], or the Leiter International Performance Scale—Third Edition [75], depending on age and expressive language development. Other instruments administered were the Quality of Life in Autism Questionnaire (QOL-A) [76] and the World Health Organization's Quality of Life Questionnaire (WHOQOL) [77] to assess separately maternal and paternal quality of life.

Blood samples from patients were collected in EDTA tubes and centrifugated within 20 min after venipuncture at 140G for 25 min at 4 °C. The platelet-rich plasma (PRP) was the collected, aliquoted and stored at -80 °C until quantification of serotonin by HPLC, as described [78]. Tempus or PAXgene tubes for RNA extraction from whole blood, and first-morning urines were also collected stored at -80 °C for experiments outside the scope of the present study (Suppl. Figure 1A).

### Data analysis

Descriptive statistics are used throughout the manuscript. Inferential statistics for auxometric parameters were performed by chi-squared tests, applying Monte Carlo significance testing (10.000 permutations) whenever more than 20% cells have an expected cell counts < 5. Serotonin blood levels were contrasted using the paired t-test to perform intrafamiliar affected vs unaffected sibling comparisons, and the Kruskal–Wallis non-parametric ANOVA followed by pairwise contrasts applying a stringent Bonferroni correction for multiple testing, to compare PMS vs iASD vs PMS unaffected siblings vs iASD unaffected siblings. In the latter case, non-parametric statistics were applied because of significant deviation of the data set from homogeneity of variance. Statistical significance was set at *p* < 0.05 and two-tail p values are applied throughout the manuscript. Data were analyzed with SPSS version 28.0 [IBM Corp. Released 2021. IBM SPSS Statistics for Windows, Version 28.0. Armonk, NY: IBM Corp].

## Results

### Demographic information and genetic findings

Demographic information and genetic characteristics at chr. 22q13.3 are presented in Table 1. Our sample includes 70 PMS patients, balanced by sex (M:F = 35:35) and aged 2 to 44 years old (mean age: 11.8 ± 9.7). Fifty-nine participants (84.3%) showed chr. 22q13.3 terminal or intragenic deletions causing haploinsufficiency of the *SHANK3* gene. Deletion size varied from a 25 kb intragenic *SHANK3* deletion, up to 9.008 Mb, but most frequently ranged from < 110 kb to 1 Mb (26/70, 37.1%) (Table 1). In sixteen cases (25.4%) the deletion involved *SHANK3* only (< 110 kb). Six patients (8.6%) carried a ring chromosome also associated with a deletion, while five patients (7.1%) carried a disruptive *SHANK3* mutation. Mosaicism was found in six (8.5%) patients (Table 1).

**Table 1:** Demographic information and genetic characteristics. (*N* = 70, unless otherwise specified)

**Variable** (sample size)		**N**	**%**
**Sex**	M	35	50.0%
	F	35	50.0%
**Age at assessment**	0–5 years	19	27.1%
	6–11 years	25	35.7%
	12–17 years	13	18.6%
	> 18 years	13	18.6%
**Chr. 22q13.3 abnormalities**	Deletion	59	84.3%
	Ring chr. with deletion	6	8.6%
	SHANK3 mutation	5	7.1%
**Deletion size in Mb** **(*N* = 63)**^a^	< 110 kb (only *SHANK3*)	16	25.4%
	110 kb-1 Mb	10	15.9%
	1.0–1.99 Mb	6	9.5%
	2.0–2.99	3	4.8%
	3.0–3.99	4	6.3%
	4.0–4.99	4	6.3%
	5.0–5.99	7	11.1%
	6.0–6.99	2	3.2%
	7.0–7.99	5	7.9%
	8.0–8.99	5	7.9%
	9.0–9.99	1	1.6%
**Mosaicism (< 70% cells)**	Present	6	8.6%
	Absent	64	91.4%

^a^Deletion size in Mb reported only for patients assessed by CGH-array

### DSM-5 diagnoses, intellectual level, and autism symptoms

The most frequent clinical DSM-5 diagnosis associated with PMS was intellectual disability, present in 69 (98.6%) cases, followed by motor coordination disorder in 65 (92.9%), ASD in 20 (28.6%), and lifetime bipolar disorder in 12 (17.1%) (Table 2). Accordingly, mean IQ or GQ was 36.43 ± 20.01, largely below normative values (see Suppl. Table 1 for the outcome of all psychodiagnostics tests administered). ADOS-2 and ADI-R scores could be collected for 39 and 47 patients, respectively. Twenty-five (64.1%) patients scored above the ADOS-2 cut-off for autism or autism spectrum (*n* = 18 and 7, respectively), while eleven patients (23.4%) met full criteria for autism on the ADI-R.

**Table 2:** DSM-5 clinical diagnoses, intellectual level and autism symptoms. (*N* = 70, unless otherwise specified)

Variable (sample size)			**N**	**%**
**DSM-5 clinical diagnoses:**	Intellectual Disability		69	98.6%
	Motor Coordination Disorder/ Dyspraxia		65	92.9%
	Autism Spectrum Disorder		20	28.6%
	Bipolar Disorder	current	10	14.3%
		lifetime	12	17.1%
	ADHD		8	11.4%
	Oppositional Defiant Disorder		2	2.9%
	Depression		1	1.4%
	Generalized anxiety, panic disorder, simple phobia		1	1.4%
	Obsessive–Compulsive Disorder		1	1.4%
**Intellectual level**	Borderline (70–79)		1	1.4%
	Cognitive disability (IQ < 70)		5	7.1%
	Not testable		64	91.4%
**DSM-5 ASD severity level** **(*N* = 20)**	Requiring support		4	20.0%
	Requiring substantial support		8	40.0%
	Requiring very substantial support		8	40.0%
**ADOS-2 diagnosis** **(*N* = 39)**	Out of the spectrum		14	35.9%
	ASD spectrum NOT autism		7	17.9%
	Autism		18	46.2%
**ADI-R B: verbal / non-verbal** **(*N* = 47)**	Non-verbal		34	72.3%
	Verbal		13	27.7%
**Motor stereotypies**	Absent		13	18.6%
	Present		57	81.4%
**Insistence on sameness**	Absent		26	37.1%
	Present		44	62.9%
**Restricted interests**	Absent		43	61.4%
	Present		27	38.6%
**Sensory hypersensitivity** **(*N* = 69)**	No hypersensitivity		47	68.1%
	Hypersensitivity to sound		18	26.1%
	Hypersensitivity to touch		2	2.9%
	Both sound and touch or other senses		2	2.9%
**Pain sensitivity**	Normal or increased		14	20.0%
	Decreased		56	80.0%
**Self-stimulation**	Absent		20	28.6%
	Visual		2	2.9%
	Auditory		3	4.3%
	Tactile		6	8.6%
	Taste/smell		21	30.0%
	Mixed (two or more sensory channels)		18	25.7%

*ADI-R *Autism Diagnostic Interview – Revised, *ADOS *Autism Diagnostic Observation Schedule, *DSM *Diagnostic and Statistical Manual of Mental Disorders

Previous research has suggested that PMS patients display less repetitive behaviors as compared to iASD [14, 27, 47]. In our sample, a high frequency of repetitive behaviors was reported by parents, especially motor stereotypies (57/70, 81.4%) and insistence on sameness (44/70, 62.9%) (Table 2). Also the child neuropsychiatrist directly observed motor and/or vocal stereotypies in 28/60 (46.7%) patients during the first visit (Suppl. Table 2). In line with these rates, 22/47 (46.8%) patients scored above the cut-off on ADI-R repetitive and stereotyped behaviors, with higher score on motor mannerism (item C3, mean: 0.98 ± 1.22) and repetitive, sensory object play (item C4, mean:1.07 ± 1.06) (Suppl. Table 1). Repetitive behaviors were also captured by RBS-R, again with higher scores on ritualistic/sameness behaviors (mean: 5.2 ± 3.9) and stereotyped behaviors (mean: 4.1 ± 3.9) (Table 3).

**Table 3:** Mean scores of tests and questionnaires administered for autism symptoms, problem behaviors, repetitive behaviors, sensory profile and adaptive behaviors

Variable	Subscale	N	Mean ± SD	Range
ADOS-2	Social Affect domain	39	11.46 ± 6.10	2–20
	Restricted and Repetitive Behaviors	39	1.59 ± 1.57	0–5
	Total Score (SA + RRB)	39	13.05 ± 7.24	2–25
ADI-R	A (anomalies in social interaction)	47	11.94 ± 7.96	0–32
	B (anomalies in communication)	47	6.98 ± 4.82	0–15
	C (restricted, repetitive and stereotyped behaviors)	47	3.02 ± 2.39	0–11
	D (anomalies in the development before 36 months)	47	4.57 ± 0.93	1–5
ABC	Irritability	51	6.67 ± 6.16	0–27
	Withdrawal	51	8.25 ± 6.35	0–27
	Stereotypic Behavior	51	3.53 ± 3.13	0–12
	Hyperactivity Or Noncompliance	51	13.14 ± 9.62	0–33
	Inappropriate Speech	51	1.69 ± 2.39	0–10
CBCL	Aggressive Behavior	44	57.66 ± 7.82	50–79
	Anxious/Depressed	44	55.34 ± 6.08	50–74
	Attention Problems	44	66.95 ± 9.82	50–90
	Rule Breaking Behavior	29	56.83 ± 6.16	50–72
	Somatic Complaints	44	58.43 ± 8.83	50–87
	Social Problems	29	69.07 ± 8.94	51–97
	Thought Problems	29	63.28 ± 9.71	50–82
	Emotionally Reactive	15	60.40 ± 8.91	50–73
	Sleeps Problems	15	58.07 ± 7.87	50–70
	Withdrawn/Depressed	44	65.89 ± 11.03	50–94
	Internalization	44	58.86 ± 10.45	27–75
	Externalization	44	56.70 ± 10.15	34–75
	Total Problems	44	62.34 ± 9.67	34–79
RBS-R	Stereotyped	51	4.06 ± 3.90	0–16
	Self-Injurious	51	2.12 ± 2.75	0–13
	Compulsive	51	1.67 ± 2.20	0–8
	Ritualistic	51	5.24 ± 3.91	0–17
	Restricted	51	2.35 ± 2.20	0–8
	Total	51	15.39 ± 10.97	0–45
SSP	Tactile sensibility	49	29.33 ± 3.94	19–35
	Taste and olfactory sensibility	49	18.24 ± 3.21	7–20
	Movement sensibility	49	12.41 ± 3.26	0–15
	Hyporeactivity	49	22.53 ± 6.75	12–35
	Auditory filtering	49	19.86 ± 3.98	8–30
	Low energy	49	20.00 ± 6.75	6–30
	Visual and auditory sensibility	49	20.04 ± 4.73	7–27
	Total	49	142.39 ± 21.16	94–190
VABS-II	Communication	52	39.52 ± 15.76	19–72
	Daily Living Skills	52	45.37 ± 17.21	20–81
	Socialization	52	49.56 ± 15.98	20–79
	Motor Skills	21	55.19 ± 17.21	1–78
	IQ composite	52	43.81 ± 14.85	19–72

*ABC *Aberrant Behaviors Checklist, *ADI-R *Autism Diagnostic Interview – Revised, *ADOS *Autism Diagnostic Observation Schedule, *CBCL *Child Behavior Checklist, *RBS-R *Repetitive Behaviors Scale – Revised, *SSP *Short Sensory Profile, *VABS *Vineland Adaptive Behavior Scale

In reference to sensory processing, reduced pain sensitivity was relatively common (56/70 = 80.0%), whereas a sizable minority of patients (22/69, 31.9%) displayed behaviors interpreted by parents as resulting from hypersensitivity to sound and/or touch (Table 2). Self-stimulatory behaviors were present in 50/70 (71.4%) patients, according to parents (Table 2). SSP total score indicated anomalies in sensory processing in 47/49 (95.9%) patients, whose parents filled in the questionnaire. Largest effects were recorded in the “Underresponsive/Seek sensations” and “Low Energy” subdomains; moreover, 26/49 (53.1%) patients showed sensory anomalies in the Auditory Filtering subdomain (Table 3; Suppl. Table 1).

### Prenatal history, birth, early postnatal behavior and feeding

Prenatal and early postnatal characteristics are summarized in Table 4. Prenatal obstetric complications were present in the history of approximately half of the sample (34/70, 48.6%), especially early bleeding during the first trimester (11/70, 15.7%). Similarly, postnatal obstetric complications occurred in 35/70 (50.0%) patients, including 16 (22.9%) who required admission to the neonatal intensive care unit. Difficulties in early feeding and gastrointestinal dysfunction were also frequent, with many parents reporting that their baby could not be breastfed (22/70, 31.4%), displayed weak sucking behavior (30/70, 42.9%), difficulty swallowing and frequent choking (18/69, 26.1%), gastrointestinal reflux during the first year of life (25/68, 35.7%), and difficulty at weaning especially with chewing semisolid foods (30/70, 42.9%) (Table 4).

**Table 4:** Prenatal history, birth, early postnatal behavior and feeding. [A] nominal variables (N and %), and [B] quantitative variables (mean ± SD, range). Sample size: *N* = 70 unless otherwise specified

A. v**ariable (sample size)**		**N**	**%**
**Spontaneous abortions** **(*N* = 65)**	0–1 spontaneous abortions	62	88.6%
	2 or more spontaneous abortions	3	4.3%
**Difficulty at conception** **(*N* = 63)**	No difficulty reported	53	75.7%
	Delayed conception (> 6 months)	9	12.9%
	In vitro fertilization	1	1.4%
**Prenatal obstetric complications**	Absent	36	51.4%
	Present	34	48.6%
	• Early bleedings (I trimester)	11	15.7%
	• Early contractions (I trimester)	2	2.9%
	• Late bleedings (III trimester)	1	1.4%
	• Late contractions (III trimester)	1	1.4%
	• Gestational diabetes	1	1.4%
	• Immune activation due to infection or allergies	1	1.4%
	• IntraUterine Growth Retardation	3	4.3%
	• Isthmic cervical incontinence	1	1.4%
	• Placenta previa	1	1.4%
	• Preterm placental calcifications	1	1.4%
	• Placental abruption	1	1.4%
	• Two or more complications	10	14.3%
**Pharmacological treatments during pregnancy**	No drug treatment	42	60.0%
	Treatment for obstetric complication	17	24.3%
	Drugs for other medical conditions	9	12.9%
	Both obstetric and medical treatments	2	2.9%
**Pregnancy duration**	Born at term (37–42 wks)	55	78.6%
	Post-term (> 42 wks or induced at 41–42 wks)	8	11.4%
	Late preterm (34- < 37 wks)	7	10.0%
**Delivery**	Eutocic	30	42.9%
	Dystocic	4	5.7%
	Labor induction	7	10.0%
	Programmed cesarean section	20	28.6%
	Emergency cesarean section	9	12.9%
**Postnatal obstetric complications**	No complications	35	50.0%
	Minor (no need for intensive care)	19	27.1%
	Major (admitted to intensive care)	16	22.9%
**Breastfeeding**	Baby was breastfed	40	57.1%
	No breastfeeding for maternal issues	8	11.4%
	No breastfeeding for baby issues	22	31.4%
**Strength in sucking milk**	Normal strength in sucking milk	40	57.1%
	Weak sucking behavior	30	42.9%
**Coordination in swallowing** **(*N* = 69)**	Coordinated swallowing	51	73.9%
	Uncoordinated swallowing	18	26.1%
**Gastroesophageal reflux** **(*N* = 68)**	Absent	43	61.4%
	Present	25	35.7%
**Weaning**	Difficulty with chewing and swallowing semisolid foods	30	42.9%
**B. Variable**	**N**	**Mean**	**Range**
Birth weight (kg)	70	3.15 ± 0.55	1.5–4.4
Birth length (cm)	65	49.35 ± 2.33	41.0–54.0
Apgar score at 1 min	61	8.64 ± 1.55	2–10
Apgar score at 5 min	59	9.46 ± 1.30	3–10
Breastfeeding duration (months)	56	7.76 ± 8.77	0.4–36

### Motor and language skills, regression, problem behaviors and adaptive functions

Motor and language development, behavioral signs/symptoms, motor coordination and sleep disorders, as reported by parents or teachers, are summarized in Table 5. Based on the acquisition of independent walking in early childhood, motor development was delayed or severely hampered in 41/70 (58.6%) cases. On average, participants were able to walk independently at a mean age of 23 months (± 13, range: 11–96). Also asymmetrical or absent crawling were observed in 36/69 (52.2%) patients. Motor coordination deficits could still be observed at the time of enrollment in as many as 54–68/70 (77.1%-97.1%) cases, depending on type of coordination (Table 5).

**Table 5:** Motor and language development, behavioral signs/symptoms, motor coordination and sleep disorders, as reported by parents or teachers. Sample size: *N* = 70 unless otherwise specified

**Variable** (sample size)		**N**	**%**
**Motor development timing**	Normal timing (walking age ≤ 18 mo)	29	41.4%
	Delayed (walking age > 18 mo)	31	44.3%
	Autonomous walking never acquired	10	14.3%
**Crawling (*N* = 69)**	Crawling present and symmetrical	33	47.8%
	Crawling present, but asymmetrical	17	24.7%
	Crawling absent	19	27.5%
**Sphincter control**	Acquired sphincter control	14	20.0%
	Not acquired yet, due to young age	6	8.6%
	Delayed (> 42 mo)	13	18.6%
	Daytime only	7	10.0%
	Never acquired	30	42.9%
**Use of pacifier (*N* = 58)**	Normal Use	31	53.4%
	Never Used	16	27.6%
	Prolonged Use (> 3 yrs)	11	19.0%
**Babbling**	Normal development (6–8 mo)	16	22.9%
	Delayed	27	38.6%
	Lost after normal development	9	12.9%
	Lost after delayed development	11	15.7%
	Never acquired	7	10.0%
**Expressive language level**	No verbal language	49	70.0%
	Words only	5	7.1%
	Words and sentences	16	22.9%
**Single words acquisition**	Normal development (≤ 18 m)	3	4.3%
	Word delay (> 18 m)	18	25.7%
	Loss after normal development	2	2.9%
	Loss after developmental delay	6	8.6%
	Never acquired	41	58.6%
**Sentences acquisition**	Normal sentence development (≤ 2 yrs)	1	1.4%
	Sentence delay (> 2 yrs)	15	21.4%
	Loss after normal development	4	5.7%
	Never acquired	49	70.0%
	Not yet acquired due to age	1	1.4%
**Storytelling skills**	Anomalous or limited storytelling skills	12	17.1%
	Lost or absent story telling	58	82.9%
**Receptive language level**	Understands—no command	19	27.1%
	- single commands	15	21.4%
	- double commands	22	31.4%
	- triple commands	14	20.0%
**Pointing**	Spontaneous pointing	9	12.9%
	Delayed pointing after intervention	31	44.3%
	Loss after normal development	2	2.9%
	Loss after delayed development	2	2.9%
	Absent pointing	26	37.1%
**History of behavioral regression**	Absent	51	72.9%
	Present	19	27.1%
**Cause of regression (*N* = 19)**	Infections & fever	3	4.3%
	Vaccination	2	2.9%
	Others	2	2.9%
	Unknown	12	17.1%
**Complications after vaccination (*N* = 68)**	No complications	59	87.2%
	Post-vaccination regression	2	2.9%
	Others (usually fever)	5	7.4%
	Vaccinations not performed	2	2.9%
**Separation anxiety in kindergarten (*N* = 68)**	No difficulties	55	80.9%
	Parental assistance required initially	6	8.8%
	Failure to continue and starting postponed	1	1.5%
	Never went to kindergarten	6	8.8%
**Behavior in kindergarten (teacher reports)**	Normal behavior	2	2.9%
	Abnormal behavior reported by teachers	62	88.6%
	Never went to kindergarten	6	8.6%
**Difficulties at elementary school onset**	No difficulties starting elementary school	2	2.9%
	Learning difficulties in elementary school	7	10.0%
	Learning and emotional difficulties	34	48.6%
	School onset postponed	8	11.4%
	Preschoolers	19	27.1%
**ADHD-like symptoms** **(parental report)**	Absent	17	24.3%
	Present	53	75.7%
	- inattention	15	21.4%
	- hyperactivity	2	2.9%
	- both	36	51.4%
**Deficient emotional self-regulation (DESR)**	Absent	30	42.9%
	Present	40	57.1%
**Self-injurious behavior**	Absent	47	67.1%
	Mild	11	15.7%
	Severe	12	17.1%
**Aggressiveness towards others**	Absent	33	47.1%
	Mild	26	37.1%
	Severe	11	15.7%
**Motor coordination deficits**	Gross movements	68	97.1%
	Fine movements	64	91.4%
	Alternating movements	54	77.1%
	Visuo-motor coordination	64	91.4%
	Bimanual coordination	66	94.3%
**Hand preference (*N* = 68)**	Right	38	55.9%
	Left	10	14.7%
	Both	13	19.1%
	Not yet acquired	7	14.7%

In reference to language development, the majority of patients (49/70 = 70.0%) never acquired or lost expressive language. At the time of enrollment, only 16/70 (22.9%) were able to pronounce simple contextualized sentences. Delayed language acquisition was commonly reported, with only three (4.3%) participants being able to express single words by 18 months and only one (1.4%) able to express a full sentence by 24 months. Receptive language was more preserved, with approximately half of participants (36/70 = 51.4%) able to understand and act a string of two–three commands (Table 5). Parents reported observing behavioral regression in 19/70 (27.1%) participants, usually without being able to associate this event to any potential trigger, except for 5/70 (7.1%) among these nineteen cases where parents temporally linked regression to an immune trigger (infection or vaccine) (Table 5).

**Fig. 1:**
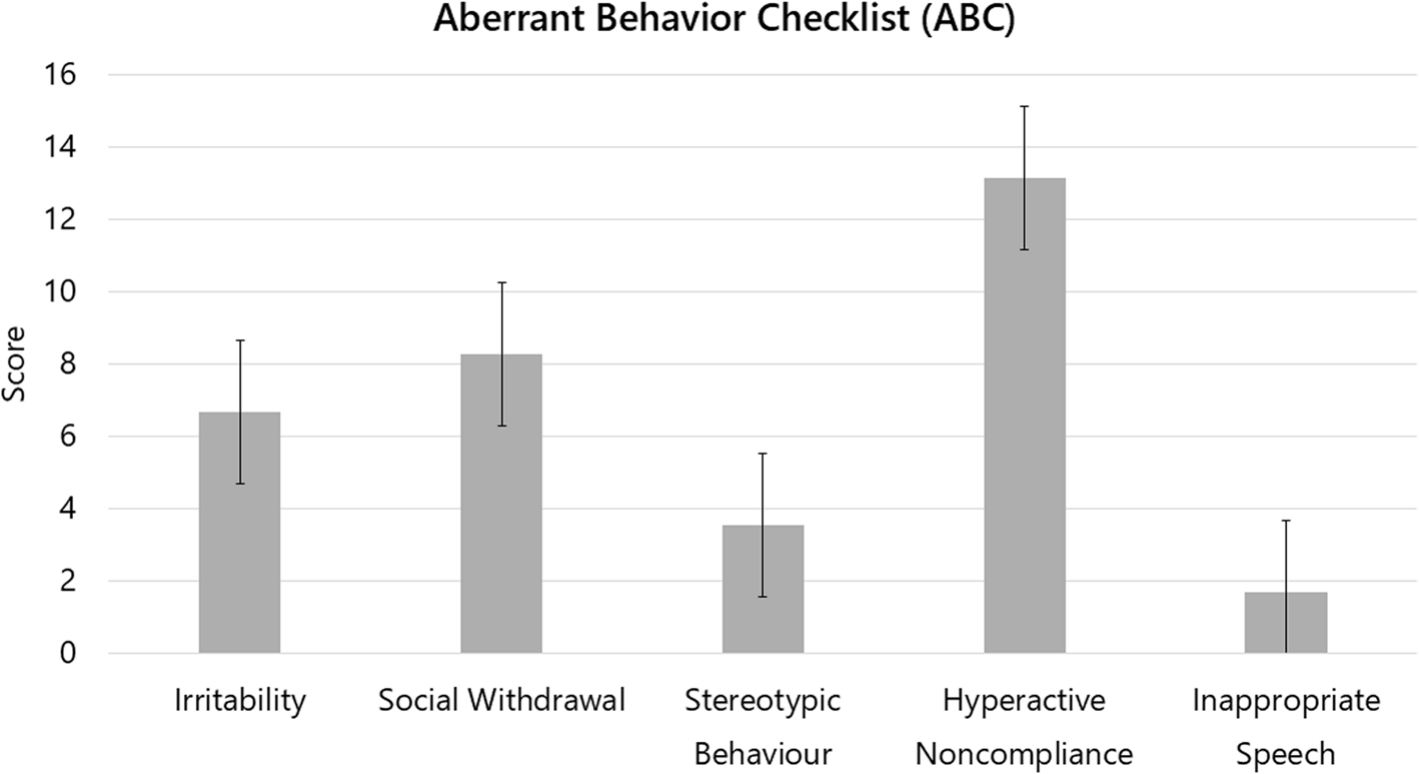
Problem behaviors measured using the Aberrant Behavior Checklist (ABC) (*n* = 51). Data reported as mean ± SEM score for each ABC subscale (*N* = 51)

**Fig. 2:**
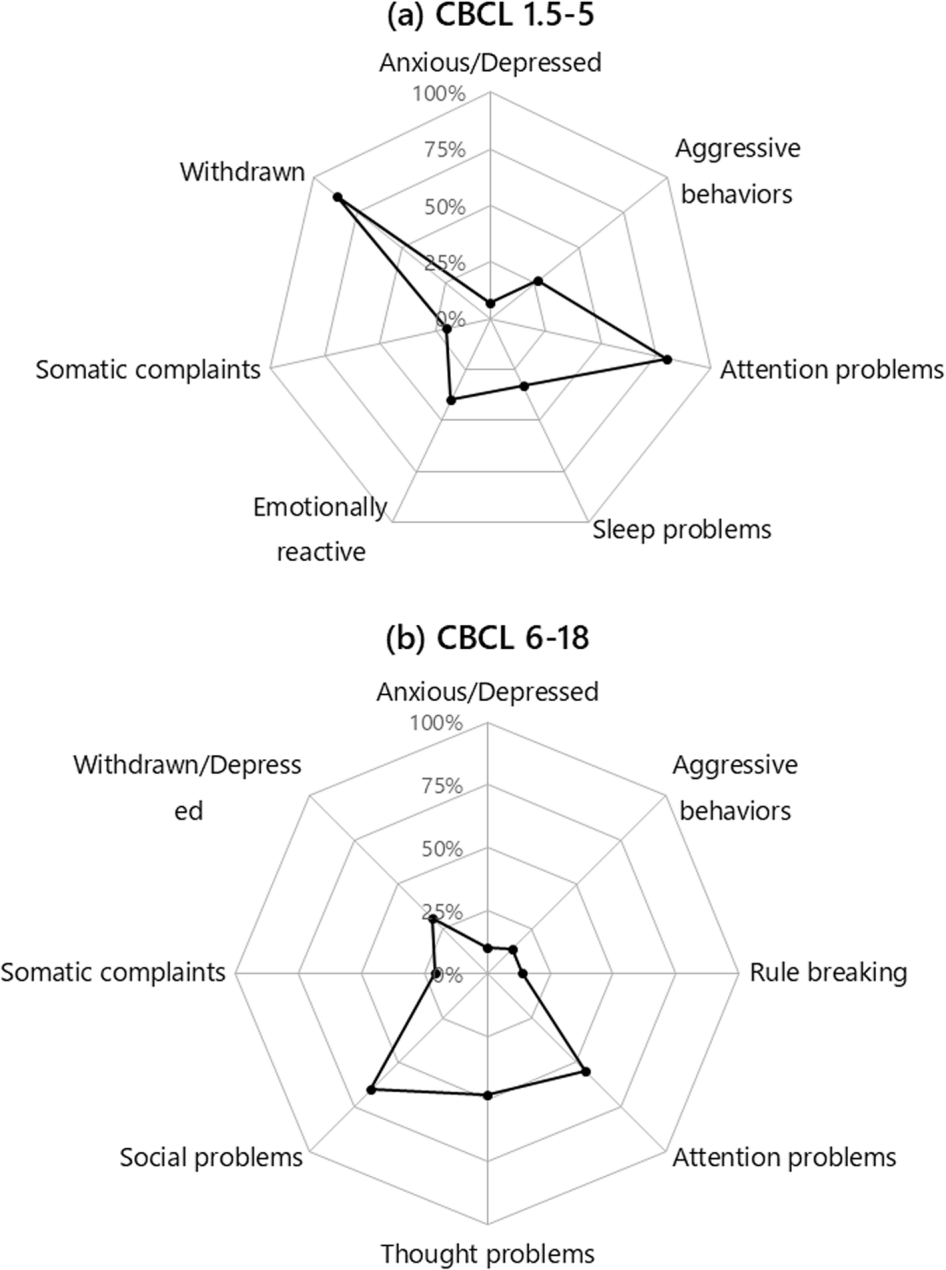
Percentage of patients above the borderline cut-off for each Child Behavior Checklist (CBCL) subscale: (**a**) Children up to 5 years of age (*n* = 15); (**b**) Children and adolescents 6–18 years old (*n* = 29)

Problem behaviors were frequent, in the forms of ADHD-like behaviors, deficient emotional self-regulation, and aggressiveness toward self or others (Table 5). Specifically, attention deficit and/or hyperactivity were reported by parents in 53/70 (75.7%) patients (Table 5). Importantly, parental reports are superimposable to accounts from direct observation by the neuropsychiatric during the intake visit, with attention deficits and hyperactivity observed in 49/61 (80.4%) and 19/61 (31.1%) cases, respectively (a complete list of behaviors directly observed by the neuropsychiatrist is provided in Suppl. Table 2). ADHD-like symptoms also emerged from questionnaires, namely ABC Hyperactive/Noncompliance (Fig. 1 and Table 3) and CBCL “Attention problems” (*n* = 44) (Fig. 2 and Table 3), with 28/44 (63.6%) participants scoring above the cut-off. Mild to severe aggressiveness towards others and self-injurious behaviors were reported by parents in 37 (52.9%) and 23 patients (32.9%), respectively. Persistent physical signs of self-injurious behavior, usually in the form of calluses in the dorsal surface of one hand, were observed by doctors in 9/60 (15.0%) patients at intake (Suppl. Table 2). CBCL Aggressive Behavior subscale, however, did not exceed the clinically significant threshold (*n* = 44; Fig. 2 and Table 4).

Predictably, major deficits in adaptive behaviors were recorded. Mean VABS-II composite score for 52 patients was 43.8, well below normative values (Table 3). Not surprisingly, lowest scores were recorded in the Communication domain (39.5 ± 15.8).

### Auxometric parameters

**Fig. 3:**
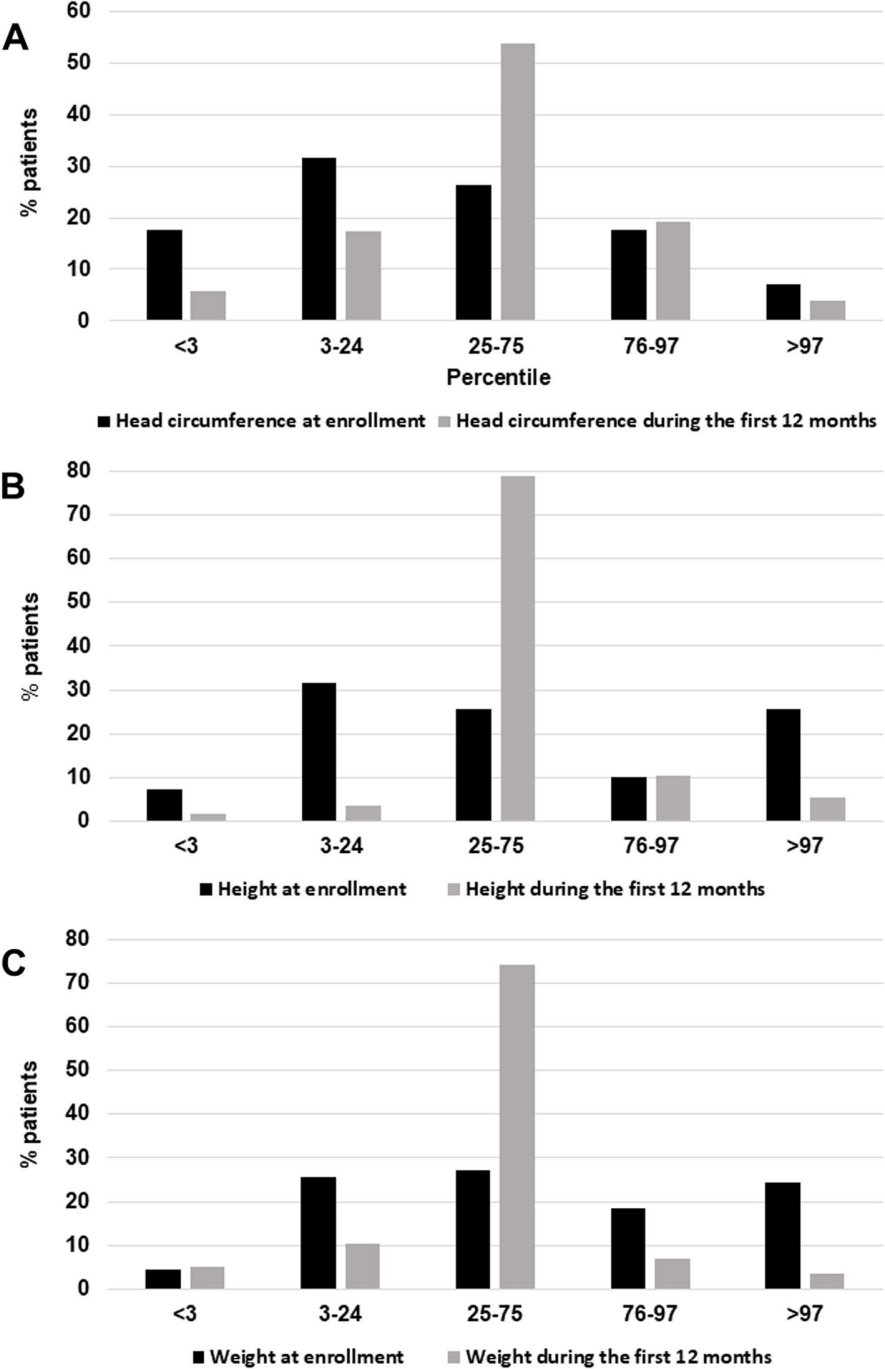
Percentile distributions of (**A**) head circumference, (**B**) height, and (**C**) weight, measured at the time of enrollment in our study (black columns) and during the first year of postnatal life (gray columns)

Height, weight and cranial circumference were measured at patient enrollment and compared with median measures collected by the pediatrician during the first year of postnatal life (Fig. 3A-C; Suppl. Table 2). Auxometric data for the first year and at enrollment were available for *N* = 52 and 57 cases for head circumference, *N* = 57 and 70 cases for height, and *N* = 58 and 70 cases for weight, respectively (Suppl. Table 3). Median first-year measures were normally distributed at all three parameters (Fig. 3A-C, gray columns). Instead, at the time of enrollment, corresponding to 11.8 ± 9.7 y.o., the distribution of head circumference displayed a leftward shift (Fig. 3A), while height and weight appeared either decreased or increased compared to early-life distributions (Fig. 3B-C, black columns). These differences are statistically significant for all three parameters, when analyzed for independent samples (Fig. 3A-C: χ^2^ = 11.2, 4 df, *p* < 0.05 for head circumference; χ^2^ = 40.8, 4 df, *p* < 1 × 10^–5^ for height; χ^2^ = 31.0, 4 df, *p* < 1 × 10^–5^ for weight). Performing intraindividual χ^2^ analyses for dependent samples exclusively on patients for whom both first year and current measures are available (*N* = 44 for head circumference, 57 for height and 58 for weight) and applying Monte Carlo significance testing (10.000 permutations), only head circumference remained statistically significance (χ^2^ = 18.4, *p* < 0.001), with height and weight yielding *p* = 0.938 and 0.878, respectively. Non-parametric analyses applying Kendall tau-b statistics yielded superimposable p-values.

Among the 44 patients for whom both past and current head circumference measures were available, the prevalence of microcephaly (i.e., < 3rd percentile) increased from 2/44 (4.5%) to 8/44 (18.2%) between the first year of life and the time of enrollment, respectively. Similarly, the number of patients falling into the “3–24 percentile” category increased from 8/44 (18.2%) to 14/44 (31.8%), respectively. Hence, altogether 12/44 (27.3%) patients display at enrollment a head circumference smaller than the size recorded by the pediatrician during the first year of neonatal life. The central category of the distribution (percentile 25–74) drops from 24 to 10 patients, losing the 12 patients displaying a deceleration in head growth and 2/44 (4.5%) patients who develop macrocephaly (i.e., > 97th percentile).

### Physical and medical issues

Physical and medical issues are summarized in Table 6, while the results of medical exams and analyses are presented in Table 7. Minor congenital dysmorphisms were observed in 35/60 (58.3%) patients (Table 6). At neurological examination, muscle hypotonia was highly prevalent (50/61, 80.0%), followed by abnormal gait or lack of autonomous walking, observed in 47/62 (75.8%) patients (Suppl. Table 4). Gastrointestinal issues were relatively frequent: 29/70 (41.4%) cases had abnormal stool, mainly chronic constipation; 11/70 (15.7%) were following a selective liquid or semi-solid diet, due to chewing and swallowing deficits; the eruption of deciduous and permanent teeth was delayed in 9/67 (13.4%) and 8/48 (16.6%) children; gastroesophageal reflux and slow gastric emptying were reported in 5/70 (7.1%). Twenty-one (30.0%) patients suffered from various allergies (Table 6). Interestingly, unless announced by prominent muscle hypotonia and developmental delay since early neonatal life, in 34/70 (48.6%) children initial motor, behavioral, and cognitive signs of PMS were surprisingly noticed by parents during or immediately after an infectious episode, especially ear-nose-throat or lower airway infections (Table 6). At the time of enrollment, sleep was abnormal in 28/70 (40.0%) cases, while as many as 45/70 (64.3%) patients suffered from current or past sleep problems, mainly difficulty falling asleep and frequent night awakenings (Table 6). Lifetime prescription of medications for sleeping disorders was reported in 29/70 (41.1%) patients, with the majority receiving melatonin (lifetime use 21/70, 30.0%; using at the time of enrollment 9/70, 12.9%) (Suppl. Tables 5A and 5B). Other pharmacological treatments, mainly second-generation antipsychotics and valproic acid, were each taken by 10/70 (14.3%) patients, whereas 6/70 (8.6%) were taking other antiepileptic drugs and 3/70 (4.3%) were taking lithium (Suppl. Table 5B). Importantly, no patient was taking selective serotonin reuptake inhibitors (SSRIs), which would have profoundly interfered with measures of serotonin levels in platelet-rich plasma. Non-pharmacological interventions provide patients and families with a holistic care plan aimed at boosting adaptive functions and improving quality of life, moving beyond medical and pharmacological interventions. A complete list of past or current non-pharmacological interventions can be found in Suppl. Table 6. Briefly, the most commonly prescribed were psychomotor training (33/70, 47.1%) and speech therapy (31/70, 44.3%). Twenty participants (28.6%) were also using Augmentative Alternative Communication (AAC) systems (Suppl. Table 6).

**Table 6:** Past and present medical issues. Sample size: *N* = 70 unless otherwise specified

**Variable** (sample size)		**N**	**%**
**Asymmetries of cranial conformation** **(*N* = 60)**	Absent	50	83.3%
	Present	10	16.6%
	• Trigonocephaly	1	1.4%
	• Dolichocephaly	1	1.4%
	• Brachycephaly	2	2.9%
	• Flattened occiput	2	2.9%
	• Frontal bossing	2	2.9%
	• Mixed (two or more)	2	2.9%
**Congenital dysmorphisms** **(*N* = 60)**	Absent	25	41.7%
	Present	35	58.3%
	• Minor dysmorphisms	26	43.3%
	• Major dysmorphisms	9	15.0%
**Decidual teeth eruption timing** **(*N* = 68)**	Early eruption (< 4 mo)	2	2.9%
	Normal eruption (4–8 mo)	56	82.4%
	Late eruption (> 8 mo)	9	13.2%
	Unknown	1	1.5%
**Permanent teeth eruption timing** **(*N* = 66)**	Early eruption (< 5 yrs)	2	3.0%
	Normal eruption (5–7 yrs)	38	57.6%
	Late eruption (> 7 yrs)	8	12.1%
	Unknown	18	27.2%
**Feeding**	Normal feeding	57	81.4%
	Selective liquid or semisolid diet	11	15.7%
	Selective solid diet	2	2.9%
**Diet**	No specific diet	52	74.3%
	Specific diet	18	25.7%
	• Gluten free	3	4.3%
	• Casein free	3	4.3%
	• Gluten and Casein free	8	11.4%
	• Other	4	5.7%
**Stool**	Normal	41	58.6%
	Constipation	21	30.0%
	Diarrhea	5	7.1%
	Alternating	3	4.3%
**Seizures**	Absent	54	77.1%
	Rare (< 1 episode every 6 mo)	13	18.6%
	Frequent	2	2.9%
	Very frequent (> once weekly)	1	1.4%
**Allergies**	Absent	49	70.0%
	Present	21	30.0%
	• Nose and Eye Allergies	7	10.0%
	• Asthma	4	5.7%
	• Skin Allergies	3	4.3%
	• Drug Allergies	3	4.3%
	• Food Allergies	2	2.9%
	• Mixed (2 or more)	2	2.9%
**Autoimmune disorder in patient**	Absent	64	91.4%
	Present	6	8.6%
**Any immune and/or allergic disease in patient**	Absent	48	68.6%
	Present	22	31.4%
**Any infectious pathology at PMS behavioral onset**	Absent	36	51.4%
	Present	34	48.6%
	• Otitis	6	8.6%
	• Upper airways and tonsils	12	17.1%
	• Gastrointestinal diseases	1	1.4%
	• Bronchitis	9	12.9%
	• Mixed (two or more)	6	8.6%
**Any other medical/surgical disorder**	Absent	42	60.0%
	Present	28	40.0%
	• Gastroesophageal reflux	4	5.7%
	• Slow gastric emptying, pseudodiarrhea	1	1.4%
	• Vesicoureteral reflux	1	1.4%
	• Hydronephrosis	1	1.4%
	• Nocturnal enuresis	1	1.4%
	• Phimosis	1	1.4%
	• Meningitis	1	1.4%
	• Cryptorchidism	1	1.4%
	• Endocrine tumor	1	1.4%
	• Ovarian polycystosis	1	1.4%
	• Febrile convulsions	1	1.4%
	• Ichthyosis	1	1.4%
	• Fatty liver disease	1	1.4%
	• Anocuteneal fistula	1	1.4%
	• Urticaria	1	1.4%
	• Klippel-Feil syndrome	1	1.4%
	• Varus foot, plantar deformity	1	1.4%
	• Neonatal hemolysis	1	1.4%
	• Acute lymphoblastic leukemia	1	1.4%
	• Mixed	6	8.6%
**Sleep disorders**	Normal sleep	25	35.7%
	Currently abnormal sleep	28	40.0%
	Previously abnormal sleep	17	24.3%
**Sleep disorder category**	Normal sleep	25	35.7%
	Sleep onset delay	6	8.6%
	Night awakening	23	32.9%
	Both sleep onset AND night awakening	13	18.6%
	Early morning awakening	2	2.9%
	Mixed (all three)	1	1.4%
**Sleep habits**	Sleeps alone in own room	35	50.0%
	Sleeps in own bed in parents' room	6	8.6%
	Starts in own bed and moves into twin bed	11	15.7%
	Sleeps in twin bed between parents	8	11.4%
	Sleeps in twin bed in place of one parent	10	14.3%

**Table 7:** Medical exams

**Variable** (Sample size)		**N**	**%**
**EEG abnormalities** **(*N* = 68)**	Normal EEG	34	50.0%
	Aspecific abnormalities	18	26.5%
	Pathological EEG	16	23.5%
**Brain imaging** **(*N* = 67)**	Normal MRI	14	20.9%
	Abnormal MRI	53	79.1%
**Tympanogram** **(*N* = 31)**	Type A (normal)	21	67.8%
	Type B (mono-ear)	3	9.6%
	Type B (bi-ears)	2	6.5%
	Type C (mono-ear)	2	6.5%
	Type C (bi-ears)	3	9.6%
**Brainstem auditory evoked potentials (BAEP)** **(*N* = 51)**	Normal	45	88.2%
	Deafness (mono-ear)	2	3.9%
	Prolonged Latency Wave (bi-ears)	3	5.9%
	Deafness (bi-ears)	1	2.0%
**EKG** **(*N* = 63)**	Normal	57	90.4%
	Abnormal	6	9.5%
**Cardiac ultrasound** **(*N* = 60)**	Normal	48	80.0%
	Patent Foramen Ovale	2	3.3%
	Others	9	15.0%
	Mixed (2 or more abnormalities)	1	16.7%
**Abdominal ultrasound** **(*N* = 65)**	Normal	27	41.5%
	Abnormal	38	58.5%
	• Kidney and urinary tract	19	29.2%
	• Liver	6	9.2%
	• Uterus	2	3.0%
	• Ovary	4	6.1%
	• Spleen	4	6.1%
	• Mixed (2 or more organs)	3	4.6%
**Kidney malformations or stones** **(*N* = 64)**	Normal	42	65.6%
	Kidney stones only	4	6.3%
	Malformations of the kidney or urinary tract	18	28.1%
**Ophthalmology visit** **(*N* = 54)**	Normal	25	46.3%
	Astigmatism	12	22.2%
	Myopia	3	5.6%
	Hyperopia	1	1.9%
	Mixed (2 or more)	4	7.4%
	Others	6	11.1%
	Strabismus	3	5.6%
**Fundus oculi** **(*N* = 53)**	Normal	48	90.6%
	Abnormal	5	9.4%
**Thelarche / pubarche** **(*N* = 61)**	Normal maturation	56	91.8%
	Premature	5	8.2%

**Fig. 4:**
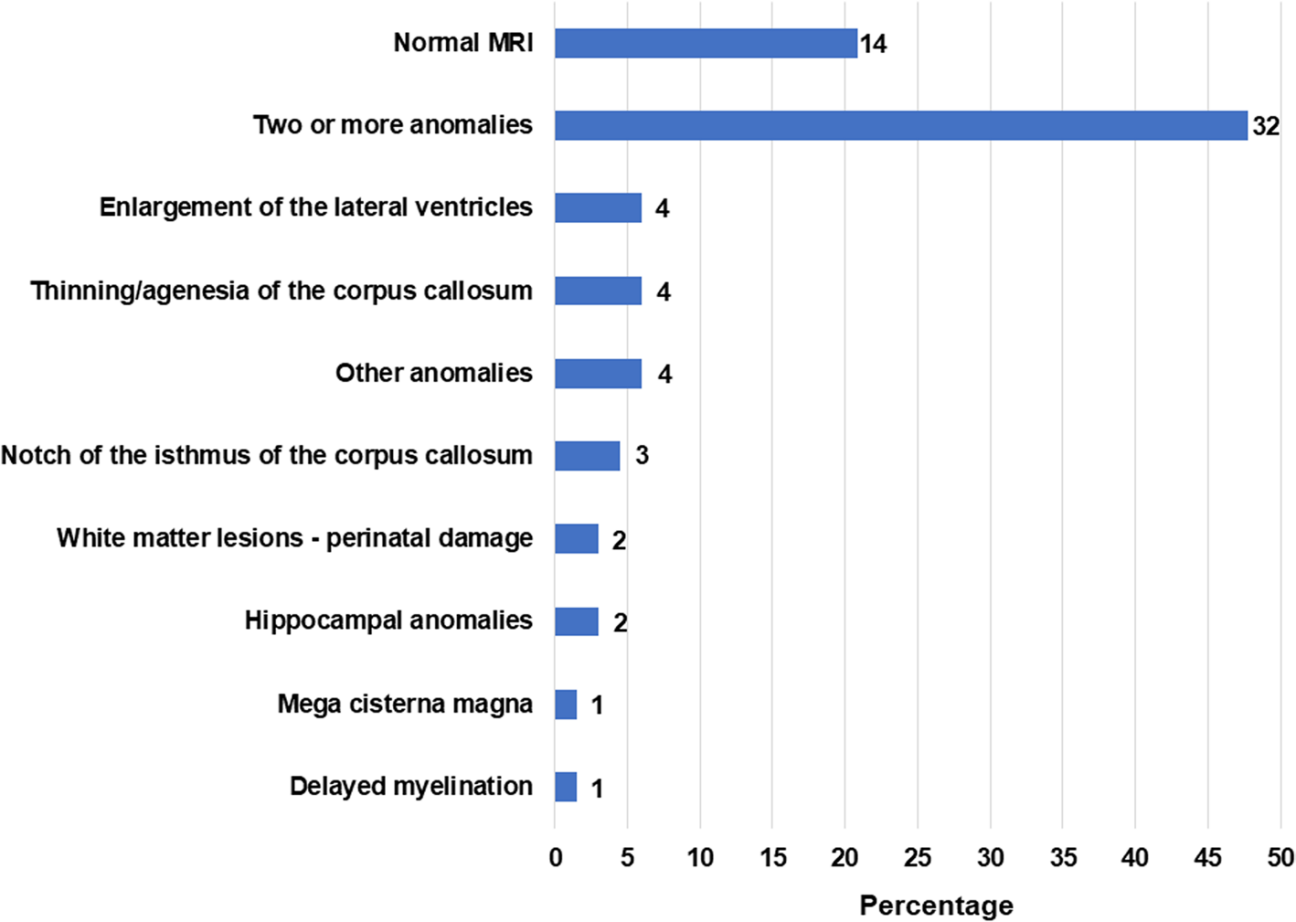
Structural brain abnormalities observed at the MRI in 67 PMS patients. Data are presented as % of patients (length of each bar referred to the X-axis), whereas numbers at the end of each bar represent patient counts

Among medical exams prescribed within the framework of our diagnostic protocol, EEG abnormalities were found in 34/68 (50.0%) patients, encompassing 18 cases with non-specific anomalies and 16 classified as “pathological” (Table 7). This outcome parallels the prevalence of seizures, which occurred in 16/70 (22.9%) patients and in most cases were occasional (i.e., less than one episode every six months) (Table 6). Brain imaging was performed in 67/70 (95.7%) patients and frequently revealed structural brain anomalies, present in 53/67 (79.1%) (Table 7). The majority of these positives displayed two or more structural brain abnormalities (Fig. 4). Abdominal ultrasound was performed in 65 patients and revealed anomalies in 38 cases (58.5%), with kidney and/or urinary tract malformations representing the most frequent positive finding (Table 7). Cardiac sonogram unveiled patent foramen ovale or congenital valve anomalies in 12/60 (20%) cases (Table 7).

### Serotonin blood levels

**Fig. 5:**
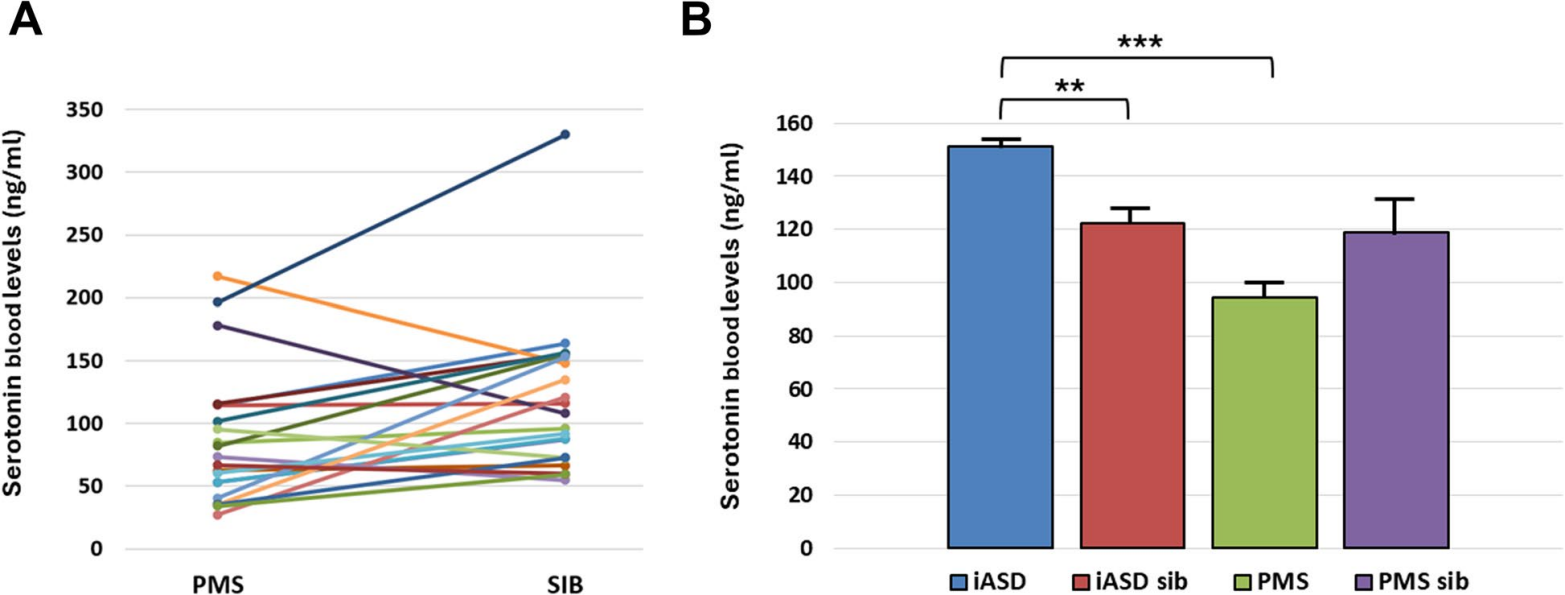
**A** Serotonin levels (ng/ml) measured in platelet-rich plasma of 21 intrafamilial PMS affected-unaffected sibling pairs; **B** Mean (± SD) serotonin levels measured in 53 PMS patients, 20 PMS unaffected siblings, 432 iASD patients, and 115 unaffected siblings of individuals with iASD. **p < 0.01, ***p < 0.001 after Bonferroni correction

A paired intrafamilial analysis was initially performed, under the hypothesis that PMS, representing a syndromic form of ASD, would have been associated with hyperserotonemia. Instead, serotonin blood levels were significantly lower in 21 PMS patients contrasted with their unaffected siblings (paired t = -2.63, 20 df, two-tail *p* < 0.05) (Fig. 5A). Indeed, the PMS-affected sibling displayed lower levels of serotonin compared to his/her unaffected sibling in 15/21 (71.4%) pairs (Fig. 5A). In order to further test this potential difference between PMS and iASD, serotonin levels measured in platelet-rich plasma of 53 PMS cases and 20 unaffected siblings, were contrasted with 432 iASD cases and 115 unaffected siblings of individuals with iASD (Fig. 5B). Serotonin blood levels differed very significantly among these four samples (K-W ANOVA: 33,065, 3 df, *p* = 3.1 × 10^–7^) (Fig. 5). PMS and iASD patients were the most distant (*p* < 0.001 after Bonferroni correction), with iASD displaying highest and PMS lowest serotonin blood levels (Fig. 5B). Unaffected siblings from iASD and PMS families displayed comparable serotonin blood levels. The difference between PMS patients and their unaffected siblings in this unpaired analysis displayed the same trend as in the paired analysis (PMS patients < PMS siblings in Fig. 5B), but did not reach significance (*P* = 0.27, n.s.). Importantly, no PMS patient was taking selective serotonin uptake inhibitors (SSRIs) and all iASD patients taking SSRIs were excluded; furthermore, only iASD cases recruited during the same period of time and at the same recruiting sites were included, in order to minimize confounding effects. This comparison confirms that PMS patients display not hyper-, but rather hyposerotonemia.

## Discussion

The aim of this study was to comprehensively describe the phenotype of a novel sample of patients with Phelan-McDermid syndrome. In addition to clinical, developmental, and psychodiagnostic parameters also assessed in several previous studies, two new areas of investigation were explored: (A) the trajectory of head and body growth, which was compared between the first year of life and the time of recruitment, and (B) serotonin blood levels, one of the most consolidated biomarkers of iASD.

Comparing median auxometric parameters recorded by the pediatrician during the first year of life and the parameters recorded at patient intake, occurring at a mean age of 11.8 years, clear differences were observed in the two distributions (Fig. 3). Head circumference, height and weight were all normally distributed during the first year of neonatal life, whereas distributions were indeed skewed at enrollment. The significant decrease in head circumference observed at the time of enrollment, compared to the first year of life, appears to be the most reliable finding, because it remains statistically significant both applying statistics for independent measures performed using all available data (*N* = 57 past vs 70 current, *p* < 0.05) and statistics for intra-individual paired measures on the smaller sample of patients for whom past and current data are both available (*N* = 47, *p* < 0.001) (Fig. 3). Instead, height and weight displayed a downward and upward shift, with some patients decelerating and others accelerating in growth over time. Interestingly, this distribution is similar to that of Rollins and Colleagues [16], who assessing 45 PMS patients found that the proportion of cases falling below the 5th and above the 95th percentile for height was significantly greater than expected [16]. Accelerated body growth has sometimes been reported as a feature of 22q13.3 deletion syndrome [18, 19]. However, further research will be necessary to verify whether the existence of two distinct subgroups for height/weight represents a real or a spurious finding, because this result did not remain significant when statistics for intra-individual paired measures were performed.

Our data clearly indicate that head growth seemingly undergoes a deceleration during childhood in approximately 25% of our PMS sample, whereas only a small minority close to 5% seemingly accelerates toward macrocephaly. This trend is not consistent with most reports of abnormal head size in PMS, pointing more often toward macrocephaly [4, 16, 17, 20]. However, prior studies made inferences based on measurements performed only at the time of patient recruitment, whereas our intra-subject comparisons enhance confidence in an actual reduction in head growth representing a much more frequent phenomenon, at least in our sample. Furthermore, this trend toward head growth deceleration over time present in approximately 25% of PMS patients clearly distinguishes PMS from iASD, whereby approximately 15.7% of autistic individuals develop macrocephaly [79]. These differences in head growth rates likely reflect different underlying neurobiologies at the level of CNS development. In fact, macrocephaly in autism is usually associated with enlarged brain volume [79]. Interestingly, a recent pooled analysis on 198 PMS patients [17] found that microcephaly was associated with smaller 22q13 deletions, leading to the suggestion that a smaller head size may reflect a smaller brain volume due to reduced myelinization, which in turn would be caused by *SHANK3* haploinsufficiency [17]. We are currently exploring the genetic underpinnings of head growth deceleration and its possible link to deletion size, characterizing its associated clinical and developmental features, and attempting to estimate the probable timing when head growth deceleration may occur during childhood.

Another intriguing result was found by performing an intrafamilial comparison between serotonin blood levels measured in PMS patients and those of their unaffected siblings. Hyperserotonemia has been consistently recorded in 22%-28% of autistic individuals [58]. Considering that PMS is often associated with autism and is regarded as a monogenic model of ASD, one might expect to find a similar result in 22q13.3 syndrome as well. On the contrary, we found that serotonin blood levels were significantly lower in PMS-affected siblings compared to their unaffected siblings (Fig. 5A). To further ensure that this was not a chance finding, we directly compared PMS vs iASD vs PMS-unaffected siblings vs iASD-unaffected siblings, demonstrating that iASD and PMS display opposite trends (Fig. 5B). Not surprisingly, the difference between PMS patients and their unaffected siblings reached significance in the paired analysis (Fig. 5A), which is statistically more powerful and more reliable, whereas it displayed the same trend but did not reach significance in the unpaired analysis, which was essentially designed to verify that PMS < iASD, but is not endowed with sufficient power when contrasting smaller samples like 53 PMS patients vs 20 unaffected siblings. Hence the unpaired analysis does not detract, but rather confirms the correctness of our observation, namely that, contrary to iASD, in PMS there is a significant reduction in serotonin blood levels. This finding is noteworthy and suggests once again that, despite the frequent presence of autistic symptoms, the underlying biology of PMS substantially differs in many aspects from the biology underlying iASD. In humans, studies on the association between serotonin blood levels and behavioral manifestations have more often focused on hyperserotonemia in iASD, which has been found associated with social difficulties, language impairment and repetitive behaviors [59–62], while hyposerotonemia has been found associated with depressive symptoms [63] and self-injurious behaviors [64], the latter heavily present in our sample. However, the significance of hyperserotonemia in iASD to this date remains elusive. In this regard, the present data are extremely interesting, because they can shed new light toward the interpretation of this biomarker, also thanks to animal models. In particular, zebrafish larvae engineered by CRISPR-Cas9 to lack the C terminus of *SHANK3* (i.e. homozygous or heterozygous *shank3abΔC* loss-of-function mutant models of Phelan-McDermid syndrome) display increased intestinal transit time due to reduced frequency of peristaltic muscular contractions [80]. This deficit in intestinal motility is associated with largely reduced numbers of serotonin-containing enteroendocrine cells in the intestinal wall, while the number of enteric neurons and serotoninergic nerve terminals is not decreased [80]. Enteroendocrine cells act as mechanosensors and chemosensors, releasing serotonin in response to mechanical stretching and/or chemical stimulation; in turn serotonin stimulates mucus secretion and peristaltic contractions, both promoting intestinal transit [81]. Importantly, the same gut enteroendocrine cells represent the primary source of the serotonin measured in the blood stream: once secreted, serotonin diffuses in the extracellular fluids throughout the gut wall, reaches the blood stream, and is captured by platelets which express the same serotonin transporter (5-HTT) and vesicular monoamine transporter (VMAT2) expressed in serotoninergic neurons of the CNS, storing serotonin in platelet vesicles and preserving it from degradation (see ref. 58 for review). Mathematical models predict that serotonin blood levels are a function of four factors: (a) platelet serotonin uptake by the 5-HTT, (b) degradation of free serotonin in the liver and lungs, (c) gut serotonin production, and (d) the volume of the gut wall [82]. Previous data have primarily linked hyperserotonemia in iASD with increased platelet serotonin uptake rates, due to common gene variants in the *ITGB3* gene and rare gene variants in the 5-HTT gene increasing serotonin uptake at the platelet membrane [61, 83]. The present data, in conjunction with the zebrafish experiments described above, underscore the influence of decreased serotonin production and release by gut enteroendocrine cells in determining hyposerotonemia in PMS. A similar mechanism may be active in some monogenic forms of ASD [84, 85], although the existence of an association between serotonin blood level and gastrointestinal symptoms in iASD is controversial [86–88]. The connection between hyposerotonemia and the gastrointestinal dysfunction frequently seen in PMS patients, on the one hand, as well as the puzzling meaning of hyposerotonemia which is also present in a minority of iASD children, will be the object of two separate ongoing investigations. Meanwhile, the unexpected discrepancy between PMS and iASD in this well-known biomarker appears endowed with great heuristic potential. Although further research is indeed necessary, interindividual differences in serotonin blood levels hold promise to reach the stage of implementation into biomarker panels for personalized medicine, contributing to dissect heterogeneity and to yield health management benefits both in iASD and in PMS.

The remaining clinical, developmental, and psychodiagnostic results replicate and extend previous description of PMS samples already present in the Literature [4, 7, 9, 22, 27], confirming that PMS typically causes developmental delay, intellectual disability, motor deficits, and severely impaired speech, in addition to increasing liability toward several medical and psychiatric comorbidities. Almost all patients fulfilled DSM-5 criteria for Intellectual Disability (99%) and Motor Coordination Disorder (93%). Expressive language was severely impaired and 70% of patients never acquired verbal language. Receptive language, on the other hand, was more preserved, but almost one third of the sample does not appear able to understand single commands (Table 5). Several problem behaviors were both reported by parents (Table 3) and observed by clinicians (Suppl. Table 2), enhancing confidence in the reliability of these data. The most common problems were ADHD-like symptoms, especially attention problems, reported in more than 70% of the sample (Table 3 and Suppl. Table 2). Hyperactivity and attention problems were also one of the most impaired domains in ABC and CBCL, respectively (Figs. 1 and 2; Table 3). Aggressiveness toward others and/or self-injurious behaviors were also frequently reported by parents (Table 5). In reference to anomalies in sensory processing, high pain threshold was reported in 80% of our sample (Table 2), in line with previous studies [4, 7, 9, 19, 20, 22]. Atypical sensory reactivity is usually found in autism as well, such that it has been included among DSM-5 criteria for ASD [50]. Nonetheless, PMS may differ from iASD in sensory profile [51]. In our sample, overall SSP total score fell within the range of probable difference, and at least two-thirds of the patients scored in the “definite difference” range (Table 3). In particular, a definite difference was found for the “Low energy”, and “Weak and Underresponsive” subscale (Table 3), which may be at least partially explained by the low muscle tone present in 50/61 (82.0%) PMS patients (Suppl. Table 4). Interestingly, scores obtained by our patients are quite similar to those reported in a previous study [51] comparing PMS and iASD sensory profile, which found that patients with PMS had more Low energy and less sensory sensitivity than iASD individuals, suggesting a different sensory profile between the two conditions.

Among medical features, a positive history of seizures and a pathological EEG recording were both present in 16/70 (22.8%) patients, a lower rate compared to approximately 40% reported in other studies [11] (Tables 6 and 7). Chronic constipation, allergies and sleep disorders were the most frequently reported (Tables 3 and 6). Other medical disorders commonly found in PMS, such as gastroesophageal reflux, were less frequent in our sample (Table 6). Sleep disorders were reported in 45 patients, and approximately 40% were taking medications for sleep, mainly melatonin (Suppl. Table 4). Only one fourth of the sample has a negative brain MRI, while almost half of the patients display multiple structural brain anomalies (Fig. 4). The prevalence of heart, kidney and urinary tract malformations was similar to previous reports [4, 7, 9, 19, 20, 22].

Obstetric complications were reported in approximately half of the sample both prenatally (especially bleedings during the I^st^ trimester) and postnatally, with 23% needing hospitalization in neonatal intensive care (Table 4). Neonatal hypotonia and early-onset motor coordination deficits were likely reflected in the difficulties in breastfeeding, sucking milk, chewing and swallowing frequently reported by parents. PMS has been previously reported to be at times characterized by a regression of previously acquired skills, which may occur immediately after a physical illness, such as an infectious disease, or at the onset of a psychiatric condition [54–56]. In our context, parents have provided two apparently contradictory sets of responses, on the one hand reporting regression in 19/70 (27.1%) children (Table 5), on the other hand reporting that “any infectious pathology at PMS behavioral onset” was observed in 34/70 (48.6%) cases (Table 6). We believe this inconsistency is only apparent and not substantial for the following reason: the term “regression” (i.e., loss of a consolidated function within a few days sometimes, but not necessarily, following a given event), is typically interpreted by parents as primarily regarding expressive language (see Table 5), and regression in language development has already been reported both in PMS [4] and in iASD [55]. Instead, the “behavioral onset” of PMS, especially in children not displaying prominent developmental delay and hypotonia since birth, involves the appearance of dysfunctional and pathological signs/symptoms previously not present, usually motor stereotypies, hyperactivity, and sleep disorders, but in some children also gaze fixation, frequent stumbling due to motor incoordination, etc. The two phenomena may only partly overlap in terms of their underlying biology and future research will greatly benefit from distinguishing more precisely between “negative” and”positive” symptoms. Regardless, the frequently observed coincidence between an infectious episode in early childhood and the worsening of behavioral, cognitive, motor, and sleep functions raises interest into possible roles of neuroinflammation as a prominent player not only iASD, but also in PMS, by precipitating the derangement of genetically-hampered neurodevelopment through acute dysfunctional activation of microglial cells and excessive synaptic pruning [89, 90]. More broadly, investigating the link between stressful life events and behavioral regression or the onset of neuropsychiatric illness will be critical to the clinical management of PMS patients. However, we must recognize that understanding and measuring reliably how”stressful” an event can be in the life of a child, adolescent, or adult with intellectual disability, motor deficits, and limited or absent expressive language represents a very challenging task.

Autistic traits were common in our sample: during the first visit, eye contact, joint attention, and reciprocal object exchange gesture were scored as “normal” by the child neuropsychiatrist in 23/60 (38.3%), 18/57 (31.6%), and 23/56 (41.1%) patients, respectively (Suppl. Table S2). However, a formal DSM-5 diagnosis of ASD was given to 20/70 (28.6%) cases (Table 2), yielding a prevalence of ASD possibly lower than that reported in other studies [4, 27]. This rate also differs substantially from the rate of “Autism” and “Autism Spectrum” diagnosed by the ADOS in a subset of our patients, collectively reaching 21/39 (53.8%). This discrepancy may stem from at least two issues. First, PMS patients are often affected by profound Intellectual Disability with severe language impairment. Many of the symptoms included among the diagnostic criteria of ASD are also common in profound Intellectual Disability accompanied by severe deficits in expressive and receptive language, including motor and vocal stereotypies. In this condition, an additional diagnosis of ASD may become highly debatable and hardly justified. In fact, the ADOS yielded higher rates, but comparable numbers of patients deserving an autism diagnosis, essentially because testable PMS patients are less affected both on the cognitive and communicative domains, and their autism can be diagnoses with greater confidence, when present. Secondly, it has been suggested that the autistic phenotype displayed by PMS patients may be somewhat different from iASD, especially in light of the fact that several studies have reported less repetitive behaviors in PMS. Repetitive and stereotyped behaviors were frequently reported by parents in our study, as was insistence on sameness (Table 2); on the contrary, fewer patients displayed restricted/limited interests and compulsive behaviors (Tables 2 and 3), compared to iASD [47]. Moreover, our RBS-R subscale and total scores are superimposable to those reported by Srivastava and Colleagues [47], and largely lower than those recorded in iASD [47]. Collectively, these results question whether repetitive and stereotyped behaviors may display different developmental trajectories and underlying neurobiologies in PMS and in iASD.

A lifetime diagnosis of bipolar disorder was found in 12 (17.1%) PMS patients. This co-morbidity is not only loaded with serious implications for the clinical management of affected PMS patients, but is also interesting from a scientific point of view, because it can be interpreted according to at least two different models: (a) first, it may represent a direct expression of the well-known polygenic overlap present among several major neuropsychiatric disorders, including bipolar, schizophrenia, and autism [91–93]; (b) secondly, abnormal neocortical wiring due to *SHANK3* synaptopathy may enhance the penetrance of genetic liability specifically towards bipolar disorder, by decreasing the efficacy of top-down control from the neocortex over limbic regions. Anecdotically, we have frequently observed familiality for bipolar disorder or for severe “anxious” depression in the first- and second-degree relatives of PMS patients with bipolar disorder, which would lend greater support to the latter model. However, a careful and reliable collection of family history information involving much larger samples of PMS patients with and without a lifetime diagnosis of bipolar disorder will be necessary to address this interesting question.

Our study has several strengths and limitations. Its main strengths consist in: (a) a thorough assessment, including detailed patients history collection, medical visits, psychodiagnostics measures, and medical exams performed both in out-patient and in-patient settings directly by the authors, implying direct observation of the patient; (b) the availability of reliable measures of height, weight, and head circumference repeatedly taken at each mandatory visit by the pediatrician during the first year of neonatal life and recorded in the child’s pediatric booklet, as prescribed by the Italian N.H.S., allowing comparison of those measures with auxometric parameters measured at the time of recruitment into this study; (c) the first analysis of serotonin blood levels both within PMS families and contrasting PMS and iASD. Its main limitations, which must be duly acknowledged, include: (a) a large prevalence of terminal chr. 22q13 deletions (89.2%) in our sample over *SHANK3* disruptive mutations, which may not reflect the ratio present in the general population. This limitation is due to the earlier implementation of CGH-array into the autism clinic compared to NGS and will likely be overcome in a few years; (b) the absence in our sample of PMS patients with interstitial chr. 22q deletions not involving SHANK3; (c) incomplete data for nine patients which were enrolled during the COVID pandemia and whose information was collected only via web without direct medical observation; (d) the lack of longitudinal observation over time by the investigators, with only parental reports providing a lifetime perspective. This limitation results in the likely underestimation of prevalence rates for conditions like gastroesophageal reflux, which often displays seasonal recurrences and must be diagnosed by the medical staff. For the same reason, prevalence rates are not being reported for signs and symptoms like lymphoedema and catatonia, which typically have a later onset in life and require long-term follow-up to be reliably diagnosed by specialized personnel.

## Conclusions

This investigation builds upon a number of published studies, providing clinical descriptions of well-characterized cohorts of PMS patients [4, 7, 9, 22, 27]. This approach is complementary to the parallel construction of patient registries, to which consenting families can themselves have access and introduce the requested information [31, 94]. The former approach provides greater reliability from direct patient observation and parental interview, as well as greater detail in the analysis of many syndromic issues; the latter approach can reach larger sample sizes, allowing to confront experimental questions difficult to address in single samples, considering PMS is a relatively rare disorder. Ideally, it would be worthwhile to conceive a collaborative phenotypic repository of published datasets in order to maintain the advantages of direct patient observation, while achieving larger sample sizes. Furthermore, only this collaborative approach would allow to investigate the possible existence of interethnic dishomogeneity in specific functional domains or developmental trajectories, possibly due to differential gene x environment interactions.

In conjunction with previous studies [4, 7, 9, 22, 27], our findings can help clinicians target appropriate patients for genetic testing, especially in clinical settings with limited resources for NGS-based gene panels or whole-exon sequencing (WES). Furthermore, the present report provides for the first time reliable evidence of PMS patient subgroups differing in head growth trajectories, which now deserve to be characterized both clinically and genetically. Meanwhile, the opposite trend in serotonin blood levels present in PMS and iASD spurs interest into the possible connection between hyposerotonemia and the gastrointestinal difficulties often observed in PMS patients. Finally, the present sample is sufficiently sized to begin investigating genotype–phenotype correlations, at least for those signs and symptoms significantly associated with deletion size. Collectively, these lines of investigation hold promise to yield results able to confer greater predictive power and to promote the clinical management of children, adolescents and adults with PMS.

## Supplementary Information

Supplementary Material 1.

Supplementary Material 2.

Supplementary Material 3.

## Data Availability

The datasets used and/or analyzed during the current study are available from the corresponding author on reasonable request.

## Data availability

Data is provided within the manuscript or supplementary information.
