# Supplementary material for: Clinical, developmental and serotonemia phenotyping of a sample of 70 Italian patients with Phelan-McDermid Syndrome
Source: J Neurodev Disord. 2024 Oct 3;16:57. doi: 10.1186/s11689-024-09572-7 (PMC11451156; doi:10.1186/s11689-024-09572-7)
Supplement: Supplementary file 1 — Supplementary Material 1. [file 11689_2024_9572_MOESM1_ESM.docx]

**Supplementary Materials**

**Clinical, developmental and serotonemia phenotyping of a sample of 70 Italian patients with Phelan-McDermid Syndrome**

**Lisa Asta^1^, Arianna Ricciardello^2^, Francesca Cucinotta^3^, Laura Turriziani^4^, Maria Boncoddo^5^, Fabiana Bellomo^6^, Jessica Angelini,^7^ Martina Gnazzo,^7^ Giulia Scandolo,^7^ Giulia Pisanò,^7^ Francesco Pelagatti,^1^ Fethia Chehbani,^1^ Michela Camia,^1^ and Antonio M. Persico^1,8*^.**

^1^Department of Biomedical, Metabolic and Neural Sciences, University of Modena and Reggio Emilia, Modena. Italy.

^2^Cantonal Psychiatric Clinic, Cantonal Socio-psychiatric Organization (O.S.C.), Repubblica e Cantone Ticino, Mendrisio, Switzerland.

^3^IRCCS Centro Neurolesi "Bonino-Pulejo", I-98124 Messina, Italy.

^4^Center for Autism “Dopo di noi”, Barcellona Pozzo di Gotto (Messina), Italy,

^5^Institute for Biomedical Research and Innovation (I.R.I.B.), National Research Council of Italy (C.N.R.), Messina, Italy.

^6^Child Neuropsychiatry Unit, “G. Martino” University Hospital, Messina (Italy).

^7^Residency Program in Child & Adolescent Neuropsychiatry, University of Modena and Reggio Emilia, Modena, Italy.

^8^Child & Adolescent Neuropsychiatry Program, Modena University Hospital, Modena, Italy.

***Correspondence:** antonio.persico@unimore.it.

ORCID: 0000-0001-8910-4479

**Supplementary Table 1.** Mean, standard deviation (SD), standard error of the mean (SEM), and range for subscales scores of all tests and questionnaires administered in this study.

| **Subscale** | **N** | **Mean** | **SD** | **SEM** | **Range** |
| --- | --- | --- | --- | --- | --- |
| **GMDS-ER_General quotient** | 26 | 24.04 | 15.326 | 3.006 | 1-56 |
| **GMDS-ER_A_Locomotor (deviation quotient)** | 24 | 30.95 | 18.103 | 3.695 | 1-79 |
| **GMDS-ER_B_Personal and social (dq)** | 24 | 22.31 | 13.088 | 2.672 | 1-54 |
| **GMDS-ER_C_Language (dq)** | 24 | 13.44 | 8.665 | 1.769 | 1-33 |
| **GMDS-ER_D_Eye and hand coordination (dq)** | 24 | 18.33 | 12.975 | 2.649 | 0-46 |
| **GMDS-ER_E_Performance (dq)** | 24 | 19.38 | 15.953 | 3.256 | 1-59 |
| **GMDS-ER_F_Pratical reasoning (dq)** | 2 | 32.70 | 10.324 | 7.300 | 25-40 |
| **VABS_communication** | 52 | 39.52 | 15.757 | 2.185 | 19-72 |
| **VABS_daily living skills** | 52 | 45.37 | 17.209 | 2.386 | 20-81 |
| **VABS_socialization** | 52 | 49.56 | 15.977 | 2.216 | 20-79 |
| **VABS_motor skills** | 21 | 55.19 | 17.209 | 3.755 | 1-78 |
| **VABS_composite IQ** | 52 | 43.81 | 14.852 | 2.060 | 19-72 |
| **ADI a1_total score** | 46 | 1.72 | 1.785 | 0.263 | 0-6 |
| **ADI a2_total score** | 46 | 3.24 | 2.861 | 0.422 | 0-10 |
| **ADI a3_total score** | 46 | 2.72 | 2.354 | 0.347 | 0-6 |
| **ADI a4_total score** | 46 | 4.43 | 2.949 | 0.435 | 0-10 |
| **ADI A_total score** | 47 | 11.94 | 7.960 | 1.161 | 0-32 |
| **ADI b1_total score** | 46 | 3.20 | 2.941 | 0.434 | 0-8 |
| **ADI b4_total score** | 46 | 3.07 | 2.533 | 0.374 | 0-6 |
| **ADI b2v_total score** | 10 | 1.60 | 1.838 | 0.581 | 0-6 |
| **ADI b3v_total score** | 10 | 1.80 | 1.619 | 0.512 | 0-4 |
| **ADI B_total score** | 47 | 6.98 | 4.821 | 0.703 | 0-15 |
| **ADI c1_total score** | 46 | 0.74 | 1.084 | 0.160 | 0-4 |
| **ADI c2_total score** | 46 | 0.30 | 0.591 | 0.087 | 0-2 |
| **ADI c3_total score** | 46 | 0.98 | 1.220 | 0.180 | 0-4 |
| **ADI c4_total score** | 46 | 1.07 | 1.063 | 0.157 | 0-4 |
| **ADI C_total score** | 47 | 3.02 | 2.391 | 0.349 | 0-11 |
| **ADI D_total score** | 47 | 4.57 | .927 | 0.135 | 1-5 |
| **ADOS-2_Social Affect domain** | 39 | 11.46 | 6.099 | 0.977 | 2-20 |
| **ADOS-2_Restricted and Repetitive Behaviors** | 39 | 1.59 | 1.568 | 0.251 | 0-5 |
| **ADOS-2_Total Score (SA+RRB)** | 39 | 13.05 | 7.244 | 1.160 | 2-25 |
| **Leiter-3_Memory** | 0 |  |  |  |  |
| **Leiter-3_Attention** | 0 |  |  |  |  |
| **Leiter-3_Total IQ Score** | 21 | 54.52 | 10.371 | 2.263 | 41-76 |
| **Wechsler scales_Verbal IQ (IQV or VCI)** | 3 | 80.00 | 24.331 | 14.048 | 64-108 |
| **Wechsler scales_Non-Verbal IQ (IQP or PRI)** | 5 | 72.20 | 46.008 | 20.575 | 21-122 |
| **WISC-IV_Working Memory Index** | 3 | 84.00 | 13.528 | 7.810 | 70-97 |
| **WISC-IV_Processing Speed Index** | 3 | 71.67 | 26.690 | 15.409 | 47-100 |
| **IQ by any intelligence scale or GMDS GQ** | 69 | 36.43 | 20.014 | 2.409 | 3-110 |
| **CGI - Severity** | 41 | 4.49 | 1.098 | 0.172 | 2-6 |
| **VAS_Social-emotional skills** | 39 | 7.051 | 1.8911 | 0.3028 | 2.0-10.0 |
| **VAS_Non-verbal communicative behaviors** | 39 | 6.692 | 2.2496 | 0.3602 | 1.0-10.0 |
| **VAS_Verbal communicative behaviors: expressive language** | 39 | 8.026 | 2.0582 | 0.3296 | 1.0-10.0 |
| **VAS_Verbal communicative behaviors: receptive language** | 39 | 6.692 | 2.0020 | 0.3206 | 1.0-10.0 |
| **VAS_Theory of mind** | 39 | 7.000 | 1.8918 | 0.3029 | 1.0-10.0 |
| **VAS_Stereotyped or repetitive movements** | 39 | 4.333 | 3.4969 | 0.5599 | 0.0-10.0 |
| **VAS_Insistence on sameness** | 39 | 4.641 | 3.0564 | 0.4894 | 0.0-9.0 |
| **VAS_Restricted interests** | 39 | 5.154 | 3.0396 | 0.4867 | 0.0-9.0 |
| **VAS_Sensory issues** | 39 | 4.410 | 3.4696 | 0.5556 | 0.0-9.0 |
| **VAS_Imitation** | 39 | 6.103 | 3.0070 | 0.4815 | 0.0-10.0 |
| **VAS_Play** | 39 | 6.923 | 2.8227 | 0.4520 | 0.0-10.0 |
| **VAS_Fine-motor skills** | 39 | 6.910 | 1.7542 | 0.2809 | 3.0-10.0 |
| **VAS_Gross-motor skills** | 39 | 6.949 | 1.9728 | 0.3159 | 1.0-10.0 |
| **VAS_Joint attention** | 39 | 6.615 | 2.5610 | 0.4101 | 0.0-10.0 |
| **VAS_Hold an object in hand** | 39 | 1.385 | 2.7968 | 0.4478 | 0.0-8.0 |
| **VAS_Enjoyment in shared play** | 39 | 6.462 | 2.6837 | 0.4297 | 0.0-10.0 |
| **CBCL_ Score of aggressive behavior** | 44 | 57.66 | 7.815 | 1.178 | 50-79 |
| **CBCL_ Score of anxious/depressed** | 44 | 55.34 | 6.084 | 0.917 | 50-74 |
| **CBCL_ Score of attention Problems** | 44 | 66.95 | 9.824 | 1.481 | 50-90 |
| **CBCL_ Score of rule breaking behavior** | 29 | 56.83 | 6.159 | 1.144 | 50-72 |
| **CBCL_ Score of somatic complaints** | 44 | 58.43 | 8.833 | 1.332 | 50-87 |
| **CBCL_ Score of social problems** | 29 | 69.07 | 8.944 | 1.661 | 51-97 |
| **CBCL_ Score of thought problems** | 29 | 63.28 | 9.706 | 1.802 | 50-82 |
| **CBCL_ Score of emotionally reactive** | 15 | 60.40 | 8.911 | 2.301 | 50-73 |
| **CBCL_ Score of sleeps problems** | 15 | 58.07 | 7.860 | 2.029 | 50-70 |
| **CBCL_ Score of withdrawn/depressed** | 44 | 65.89 | 11.025 | 1.662 | 50-94 |
| **CBCL_ Internalization score** | 44 | 58.86 | 10.449 | 1.575 | 27-75 |
| **CBCL_ Externalization score** | 44 | 56.70 | 10.149 | 1.530 | 34-75 |
| **CBCL_ Total problems score** | 44 | 62.34 | 9.698 | 1.462 | 34-79 |
| **TRF_Score of aggressive behavior** | 31 | 60.90 | 6.024 | 1.082 | 50-72 |
| **TRF_Score of anxious/depressed** | 31 | 56.06 | 5.105 | 0.917 | 50-70 |
| **TRF_Score of attention Problems** | 31 | 65.26 | 9.227 | 1.657 | 50-88 |
| **TRF_Score of rule breaking behavior** | 21 | 56.29 | 6.018 | 1.313 | 50-69 |
| **TRF_Score of somatic complaints** | 31 | 57.68 | 8.175 | 1.468 | 50-75 |
| **TRF_Score of social problems** | 21 | 65.48 | 5.419 | 1.182 | 53-76 |
| **TRF_Score of thought problems** | 21 | 64.71 | 9.639 | 2.103 | 50-85 |
| **TRF_Score of emotionally reactive** | 10 | 66.90 | 6.262 | 1.980 | 52-74 |
| **TRF_Score of withdrawn/depressed** | 31 | 62.61 | 11.392 | 2.046 | 50-89 |
| **TRF_Internalization score** | 31 | 60.19 | 9.042 | 1.624 | 36-72 |
| **TRF_Externalization score** | 31 | 60.68 | 8.228 | 1.478 | 34-73 |
| **TRF_Total problems score** | 31 | 63.42 | 8.111 | 1.457 | 41-77 |
| **QoLA_A score mother** | 48 | 90.21 | 14.048 | 2.028 | 63-122 |
| **QoLA_B score mother** | 49 | 70.47 | 15.663 | 2.238 | 39-100 |
| **QoLA_A_item 28 score mother** | 44 | 2.36 | 1.014 | 0.153 | 1-5 |
| **QoLA_A score father** | 47 | 90.96 | 14.443 | 2.107 | 64-129 |
| **QoLA_B score father** | 46 | 74.26 | 15.141 | 2.232 | 40-100 |
| **QoLA_A_item 28 score father** | 43 | 2.67 | 1.128 | 0.172 | 1-5 |
| **WHOQOL_total score mother** | 50 | 87.66 | 11.984 | 1.695 | 66-119 |
| **WHOQOL_total score father** | 45 | 90.49 | 12.380 | 1.845 | 59-115 |
| **SSP_score of tactile sensibility section*** | 49 | 29.33 | 3.944 | 0.563 | 19-35 |
| **SSP_Taste and olfactory sensibility section** | 49 | 18.24 | 3.205 | 0.458 | 7-20 |
| **SSP_Movement sensibility section** | 49 | 12.41 | 3.259 | 0.466 | 0-15 |
| **SSP_Hyporeactivity section** | 49 | 22.53 | 6.746 | 0.964 | 12-35 |
| **SSP_Auditory filtering section** | 49 | 19.86 | 3.979 | 0.568 | 8-30 |
| **SSP_Low energy section** | 49 | 20.00 | 6.752 | 0.965 | 6-30 |
| **SSP_Visual and auditory sensibility section** | 49 | 20.04 | 4.730 | 0.676 | 7-27 |
| **SSP_Total score** | 49 | 142.39 | 21.160 | 3.023 | 94-190 |
| **Aberrant Behavior Checklist (ABC)_irritability** | 51 | 6.67 | 6.160 | 0.863 | 0-27 |
| **ABC_withdrawal** | 51 | 8.25 | 6.352 | 0.890 | 0-27 |
| **ABC_stereotypic behavior** | 51 | 3.53 | 3.126 | 0.438 | 0-12 |
| **ABC_hyperactivity or noncompliance** | 51 | 13.14 | 9.623 | 1.347 | 0-33 |
| **ABC_inappropriate speech** | 51 | 1.69 | 2.387 | 0.334 | 0-10 |
| **RBS_Repetitive behavior scale_revised_stereotyped behavior** | 51 | 4.06 | 3.896 | 0.546 | 0-16 |
| **RBS_self injurious subscale** | 51 | 2.12 | 2.747 | 0.385 | 0-13 |
| **RBS_compulsive subscale** | 51 | 1.67 | 2.197 | 0.308 | 0-8 |
| **RBS_ritualistic behavior subscale** | 51 | 5.24 | 3.912 | 0.548 | 0-17 |
| **RBS_restricted behavior subscale** | 51 | 2.35 | 2.198 | 0.308 | 0-8 |
| **RBS_total_score** | 51 | 15.39 | 10.971 | 1.536 | 0-45 |
| **RBS_ global rating score** | 33 | 35.42 | 27.482 | 4.784 | 1-80 |
| **RBS_stereotyped behavior subscale_Number endorsed** | 51 | 2.67 | 2.007 | 0.281 | 0-8 |
| **RBS_self injurious subscale_Number endorsed** | 51 | 1.33 | 1.532 | 0.215 | 0-5 |
| **RBS_compulsive subscale_Number endorsed** | 51 | 1.14 | 1.281 | 0.179 | 0-4 |
| **RBS_ritualistic behavior subscale_Number endorsed** | 51 | 3.61 | 2.324 | 0.325 | 0-10 |
| **RBS_restricted behavior subscale_Number endorsed** | 51 | 1.39 | 1.168 | 0.163 | 0-3 |
| **RBS_Total Number endorsed** | 51 | 10.08 | 5.979 | 0.837 | 0-27 |
| **SCQ_total score** | 43 | 15.33 | 4.824 | 0.736 | 5-23 |

**Supplementary Table 2.** Behaviors directly observed by the neuropsychiatrist during the intake visit.

| **Variable** (sample size) | | **N** | **Percent** |
| --- | --- | --- | --- |
| **Hyperactivity**  **(N=61)** | Absent | 42 | 68.9% |
|  | Present | 18 | 29.5% |
|  | Extreme | 1 | 1.6% |
| **Attention deficit**  **(N=61)** | Absent | 12 | 19.7% |
|  | Present | 49 | 80.3% |
| **Oppositional traits**  **(N=61)** | Absent | 40 | 65.6% |
|  | Present | 21 | 34.4% |
| **Play behaviors**  **(N=60)** | Absent | 12 | 20.0% |
|  | Disorganized play | 2 | 3.3% |
|  | Manipulative/Object Play | 20 | 33.4% |
|  | Imitation Play | 8 | 13.3% |
|  | Pretend Play | 18 | 30.0% |
| **Eye contact**  **(N=60)** | Normal | 23 | 38.3% |
|  | Inconsistent | 31 | 51.7% |
|  | Absent or very rare | 6 | 10.0% |
| **Reciprocal object exchange gesture**  **(N=56)** | Complete | 23 | 41.1% |
|  | Incomplete | 7 | 12.5% |
|  | Absent | 24 | 42.8% |
|  | Not testable | 2 | 3.6% |
| **Joint attention**  **(N=57)** | Complete | 18 | 31.6% |
|  | Incomplete | 11 | 19.3% |
|  | Absent | 27 | 47.4% |
|  | Not testable | 1 | 1.7% |
| **Imitation ability**  **(N=57)** | Present | 29 | 50.9% |
|  | Absent | 26 | 45.6% |
|  | Not testable | 2 | 3.5% |
| **Stereotyped behaviors (motor and/or vocal)**  **(N=60)** | Absent | 32 | 53.4% |
|  | Present | 26 | 43.3% |
|  | Intense, continuous or extreme | 2 | 3.3% |
| **Self-stimulation: main sensory channel**  **(N=61)** | No self-stimulation | 40 | 65.6% |
|  | Taste self-stimulation | 14 | 23.0% |
|  | Tactile self-stimulation | 6 | 9.8% |
|  | Auditory self-stimulation | 1 | 1.6% |
| **Self-stimulation: secondary sensory channel (N=61)** | None | 58 | 95.1% |
|  | Taste self-stimulation | 3 | 4.9% |
| **Signs of self-aggressiveness on the skin (N=60)** | Absent | 51 | 85.0% |
|  | Present | 9 | 15.0% |

**Supplementary Table 3**. Auxometric parameters measured at the time of patient enrollment and during the first 12 months of postnatal life.

|  | **Percentile** | **N** | **%** |
| --- | --- | --- | --- |
| **Head circumference at enrollment (N=57)** | <3 | 10 | 17.5 |
|  | 3-24 | 18 | 31.6 |
|  | 25-75 | 15 | 26.3 |
|  | 76-97 | 10 | 17.6 |
|  | >97 | 4 | 7.0 |
| **Head circumference during the first 12 months**  **(N=52)** | <3 | 3 | 5.8 |
|  | 3-24 | 9 | 17.3 |
|  | 25-75 | 28 | 53.9 |
|  | 76-97 | 10 | 19.2 |
|  | >97 | 2 | 3.8 |
| **Height**  **(N=70)** | <3 | 5 | 7.2 |
|  | 3-24 | 22 | 31.4 |
|  | 25-75 | 18 | 25.7 |
|  | 76-97 | 7 | 10.0 |
|  | >97 | 18 | 25.7 |
| **Height during the first 12 months**  **(N=57)** | <3 | 1 | 1.8 |
|  | 3-24 | 2 | 3.5 |
|  | 25-75 | 45 | 78.9 |
|  | 76-97 | 6 | 10.5 |
|  | >97 | 3 | 5.3 |
| **Weight**  **(N=70)** | <3 | 3 | 4.3 |
|  | 3-24 | 18 | 25.7 |
|  | 25-75 | 19 | 27.1 |
|  | 76-97 | 13 | 18.6 |
|  | >97 | 17 | 24.3 |
| **Weight during the first 12 months**  **(N=58)** | <3 | 3 | 5.2 |
|  | 3-24 | 6 | 10.3 |
|  | 25-75 | 43 | 74.1 |
|  | 76-97 | 4 | 6.9 |
|  | >97 | 2 | 3.5 |

**Supplementary Table 4.** Neurological examination performed by the neuropsychiatrist during the intake visit.

| **Variable** (sample size) | | **N** | **Percent** |
| --- | --- | --- | --- |
| **Muscle tone**  **(N=61)** | Normal | 8 | 13.1% |
|  | Hypotonia | 50 | 82.0% |
|  | Elastic hypertonus | 3 | 4.9% |
| **Muscle size**  **(N=59)** | Normal | 49 | 83.1% |
|  | Hypotrophic | 9 | 15.2% |
|  | Hypertrophic | 1 | 1.7% |
| **Muscle strength**  **(N=56)** | Normal | 43 | 76.8% |
|  | Reduced | 13 | 23.2% |
| **Cerebellar signs**  **(N=61)** | Absent | 3 | 4.9% |
|  | Present | 3 | 4.9% |
|  | Not testable | 55 | 90.2% |
| **Romberg sign**  **(N=60)** | Negative | 5 | 8.3% |
|  | Positive | 1 | 1.7% |
|  | Not testable | 54 | 90.0% |
| **Walking**  **(N=62)** | Normal autonomous walking | 15 | 24.2% |
|  | Abnormal autonomous walking | 39 | 62.9% |
|  | Non-autonomous walking | 5 | 8.1% |
|  | Absent | 3 | 4.8% |
| **Walking on toes and heels**  **(N=58)** | Absent | 2 | 3.5% |
|  | Present | 6 | 10.3% |
|  | Not testable | 50 | 86.2% |
| **Flat feet**  **(N=59)** | Absent | 36 | 61.0% |
|  | Present | 23 | 39.0% |
| **Tendon reflexes - lower limbs**  **(N=58)** | Normal | 23 | 39.6% |
|  | Hypoelicitable or absent | 20 | 34.5% |
|  | Hyperelicitable | 15 | 25.9% |
| **Tendon reflexes - upper limbs**  **(N=58)** | Normal | 29 | 50.0% |
|  | Hypoelicitable or absent | 18 | 31.0% |
|  | Hyperelicitable | 11 | 19.0% |
| **Static balance**  **(N=59)** | Normal | 7 | 11.9% |
|  | Abnormal | 27 | 45.8% |
|  | Not testable | 25 | 42.3% |
| **Dynamic balance**  **(N=61)** | Normal | 3 | 4.9% |
|  | Abnormal | 48 | 78.7% |
|  | Not testable | 10 | 16.4% |

**Supplementary Table 5.** (A) Lifetime prescription history for sleep disorders. (B) Pharmacological treatments at the time of the first visit.

Sample size: N=70

**A**

|  |  | **N** | **%** |
| --- | --- | --- | --- |
| **Lifetime drug therapy for sleep disorders** | None | 41 | 58.6% |
|  | Melatonin | 16 | 22.9% |
|  | Other psychoactive drugs (SGAs, antidepressants, etc) | 6 | 8.6% |
|  | Both melatonin and other psychoactive drugs | 5 | 7.1% |
|  | Nutraceutics without melatonin | 1 | 1.4% |
|  | Benzodiazepines | 1 | 1.4% |

**B**

| **Pharmacological agents used at enrollment** | **N** | **%** |
| --- | --- | --- |
| **Second generation antipsychotics (SGAs)** | 10 | 14.3% |
| **Sodium valproate** | 10 | 14.3% |
| **Melatonin** | 9 | 12.9% |
| **Other antiepileptics** | 6 | 8.6% |
| **Lithium** | 3 | 4.3% |
| **Selective serotonin reuptake inhibitors (SSRIs)** | 0 | - |

**Supplementary Table 6.** Past and current non-pharmacological therapeutic interventions

Sample size: N=70 unless otherwise specified.

| **Variable** (Sample size) | | **N** | **%** |
| --- | --- | --- | --- |
| **Psychomotor training** | never | 4 | 5.7% |
|  | past | 33 | 47.1% |
|  | current | 33 | 47.1% |
| **Speech therapy** | never | 17 | 24.3% |
|  | past | 22 | 31.4% |
|  | current | 31 | 44.3% |
| **ESDM** | never | 69 | 98.6% |
|  | current | 1 | 1.4% |
| **CBT** | never | 65 | 92.9% |
|  | past | 2 | 2.9% |
|  | current | 3 | 4.3% |
| **Behavioral therapy (ABA)** | never | 60 | 85.7% |
|  | past | 1 | 1.4% |
|  | current | 9 | 12.9% |
| **AAC (PECS or signs) (N=69)** | never | 42 | 60.9% |
|  | past | 7 | 10.1% |
|  | current | 20 | 29.0% |
| **Animal-Assisted Intervention/ Horse therapy** | never | 56 | 80.0% |
|  | past | 4 | 5.7% |
|  | current | 10 | 14.3% |
| **Parent Training** | never | 69 | 98.6% |
|  | current | 1 | 1.4% |
| **Psychomotor training in water** | never | 59 | 84.3% |
|  | past | 2 | 2.9% |
|  | current | 9 | 12.9% |
| **Psychopedagogical intervention** | never | 66 | 94.3% |
|  | past | 1 | 1.4% |
|  | current | 3 | 4.3% |
| **Occupational therapy** | never | 61 | 87.1% |
|  | past | 1 | 1.4% |
|  | current | 8 | 11.4% |
| **Sport (longest practice)** | No Sport | 51 | 72.9% |
|  | Water Sport | 13 | 18.6% |
|  | Dancing | 1 | 1.4% |
|  | Others | 3 | 4.3% |
|  | Two or more sports | 2 | 2.9% |

AAC: Augmentative Alternative Communication; ABA: Applied Behavioral Analysis; CBT: Cognitive Behavioral Therapy; ESDM: Early Start Denver Model; PECS: Picture Exchange Communication System.

**Supplementary Figure legends**

**Supplementary Figure 1.** (A) Medical protocol, including visits and exams performed as inpatients and as outpatients; and (B) psychodiagnostic protocol, subdivided into the series of meetings held with patients and families.
