# Supplementary figures and images for: Clinical, developmental and serotonemia phenotyping of a sample of 70 Italian patients with Phelan-McDermid Syndrome
Source: J Neurodev Disord. 2024 Oct 3;16:57. doi: 10.1186/s11689-024-09572-7 (PMC11451156; doi:10.1186/s11689-024-09572-7)

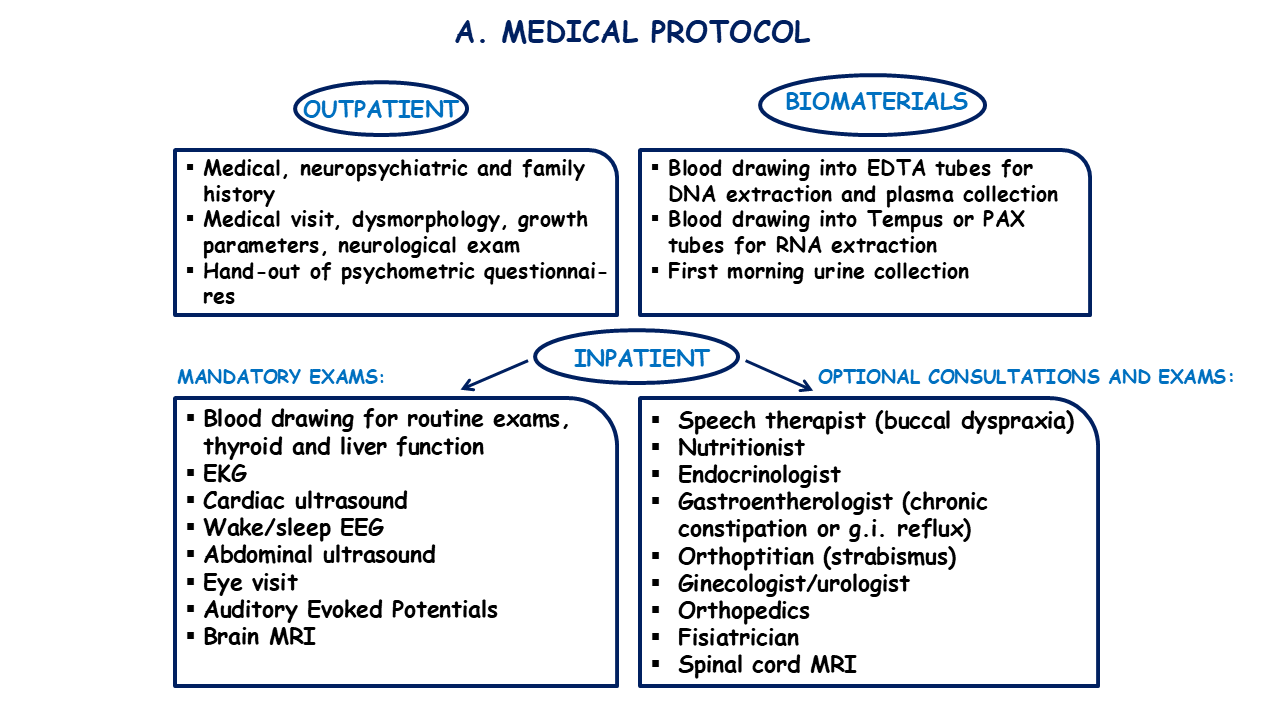

Supplement: Supplementary file 2 — Supplementary Material 2. [file 11689_2024_9572_MOESM2_ESM.tif]

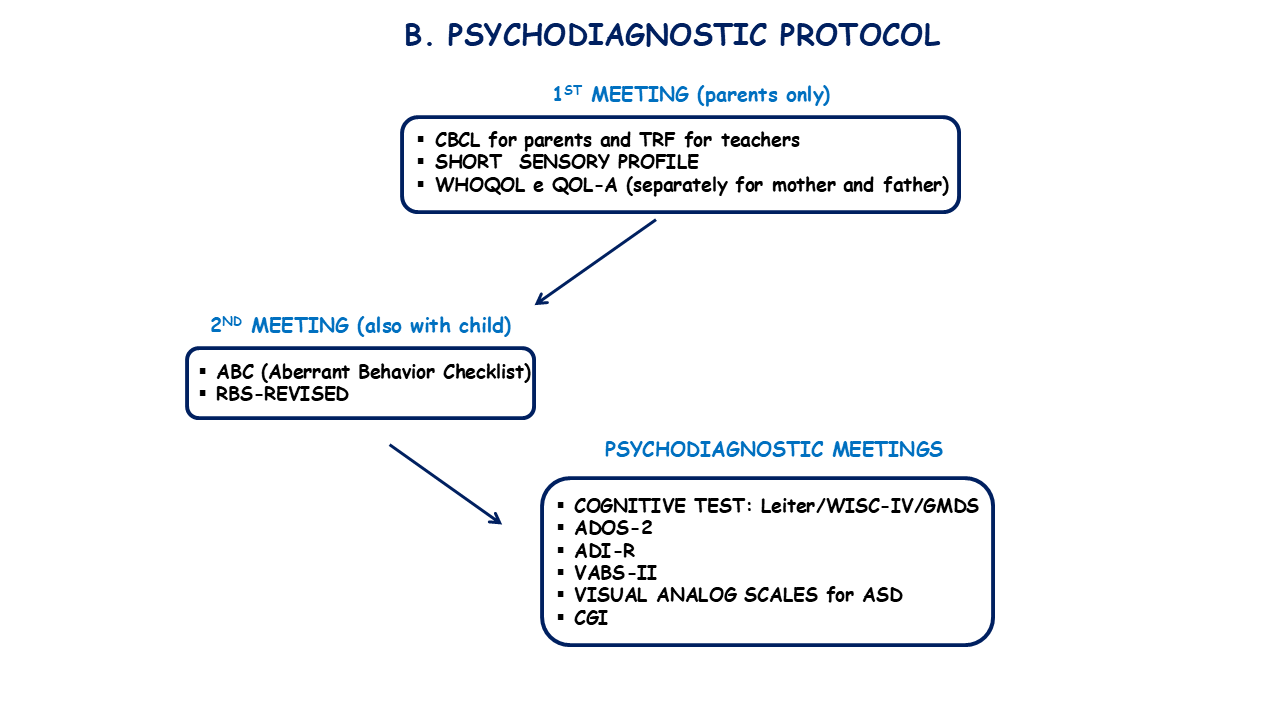

Supplement: Supplementary file 3 — Supplementary Material 3. [file 11689_2024_9572_MOESM3_ESM.tif]
